# Regulation of the ER stress response by a mitochondrial microprotein

**DOI:** 10.1038/s41467-019-12816-z

**Published:** 2019-10-25

**Authors:** Qian Chu, Thomas F. Martinez, Sammy Weiser Novak, Cynthia J. Donaldson, Dan Tan, Joan M. Vaughan, Tina Chang, Jolene K. Diedrich, Leo Andrade, Andrew Kim, Tong Zhang, Uri Manor, Alan Saghatelian

**Affiliations:** 1 The Salk Institute for Biological Studies, Clayton Foundation Laboratories for Peptide Biology, 10010N. Torrey Pines Rd, La Jolla, CA 92037 USA; 2 The Salk Institute for Biological Studies, Waitt Advanced Biophotonics Center, 10010N. Torrey Pines Rd, La Jolla, CA 92037 USA

**Keywords:** Mitochondrial proteins, Protein-protein interaction networks

## Abstract

Cellular homeostasis relies on having dedicated and coordinated responses to a variety of stresses. The accumulation of unfolded proteins in the endoplasmic reticulum (ER) is a common stress that triggers a conserved pathway called the unfolded protein response (UPR) that mitigates damage, and dysregulation of UPR underlies several debilitating diseases. Here, we discover that a previously uncharacterized 54-amino acid microprotein PIGBOS regulates UPR. PIGBOS localizes to the mitochondrial outer membrane where it interacts with the ER protein CLCC1 at ER–mitochondria contact sites. Functional studies reveal that the loss of PIGBOS leads to heightened UPR and increased cell death. The characterization of PIGBOS reveals an undiscovered role for a mitochondrial protein, in this case a microprotein, in the regulation of UPR originating in the ER. This study demonstrates microproteins to be an unappreciated class of genes that are critical for inter-organelle communication, homeostasis, and cell survival.

## Abstract (web summary)

Cells trigger an unfolded protein response (UPR) in the endoplasmic reticulum, but its regulation by mitochondria is unclear. Here, the authors report a 54-residue microprotein PIGBOS that participates in inter-organelle contact between the endoplasmic reticulum and the mitochondria and may regulate UPR.

## Introduction

The term microproteins refers to peptides and small proteins translated from small open reading frames (smORFs)^1,2^. Advances in genomics and proteomics technologies reveal that mammalian genomes harbor hundreds to thousands of previously unannotated microprotein-coding smORFs^3–5^. As a large and completely unstudied fraction of the genome, assignment of functions to smORFs and microproteins represents a major opportunity to gain new insights into biology. Only a handful of smORFs and microproteins have been characterized so far^1,2^. For example, several muscle-specific smORFs have revealed new pathways that control muscle performance and development^6,7^, and a microprotein called CYREN regulates DNA repair pathway choice during the cell cycle^8^. It is likely that many other key cellular processes are also mediated by uncharacterized microproteins and the discovery and characterization of smORFs and microproteins is an important research endeavor.

Cells routinely encounter stress that negatively impacts cell health and function. The unfolded protein response (UPR) is a fundamental pathway in eukaryotes that is triggered by the onset of endoplasmic reticulum (ER) stress resulting from the presence of unfolded proteins in the ER lumen^9,10^. Stress-responsive genes, proteins, and pathways provide a cellular mechanism to cope with this stress and return cells to homeostasis. There are three primary branches of the UPR pathway and each pathway is mediated by a different ER protein: IRE1, PERK, or ATF6^11^. Activation of these proteins during UPR initiates signals at the ER that slow down protein expression, increase protein folding, and upregulate degradation of unfolded proteins^9,10^. If these steps fail to return the cell to homeostasis and prolong activation of UPR, the cells will undergo apoptosis^12,13^. Understanding UPR regulation has implications for human health as dysregulation of UPR signaling is thought to underlie several prevalent diseases^14,15^. Here, we characterize a microprotein called PIGBOS and reveal a role for a mitochondrial protein in UPR signaling.

## Results

### PIGBOS is a conserved microprotein

**Fig. 1:**
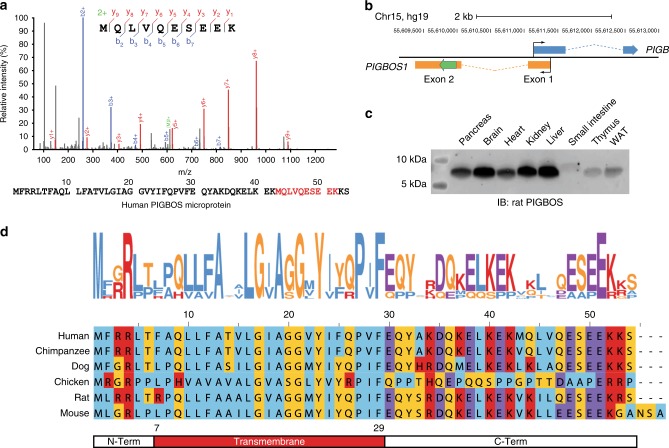
PIGBOS is a conserved microprotein. **a** Detection of a unique PIGBOS tryptic peptide by proteomics (MS/MS spectrum as shown) and the entire 54 amino acid human PIGBOS microprotein with detected tryptic peptide (red). **b** *PIGBOS1* gene contains two exons and is located on the opposite strand of the *PIGB* gene on chromosome 15. The PIGBOS protein coding sequence (CDS) in exon 2 is highlighted in green. **c** Western blot of rat tissues detects endogenous PIGBOS microprotein expression. **d** A logo plot generated from the sequence alignment of PIGBOS microprotein from multiple species reveals conserved amino acids including the transmembrane region between amino acids 7–29 (red) flanked by a N-terminal region (N-Term, aa 1–6) and a C-terminal region (C-Term, aa 30–54)

During proteomic searches for microproteins, we identified a tryptic peptide, MQLVQESEEK, from the human 54-amino acid PIGB opposite strand 1 (PIGBOS) microprotein (Fig. 1a), providing experimental evidence for PIGBOS translation. *PIGBOS* obtained its name because it is on the opposite strand of the phosphatidylinositol glycan anchor biosynthesis class B (*PIGB*) gene (Fig. 1b). The PIGBOS transcript consists of two exons and has three splice isoforms with slight differences in the first exon, but the second exon that contains the entire PIGBOS smORF is the same (Supplementary Fig. 1a). RNA-Seq and Ribosome profiling datasets provide evidence of PIGBOS expression and translation in three different human cell lines (Supplementary Fig. 1a). To further confirm whether the PIGBOS smORF is translated to produce a stable microprotein, we raised antibodies against human and rat PIGBOS. Western blot analysis of numerous human cell lines (Supplementary Fig. 1b) and rat tissues (Fig. 1c) readily detected PIGBOS, demonstrating PIGBOS to be a widely expressed, stable microprotein. PIGBOS is uncharacterized, but sequence conservation and a positive PhyloCSF score^16^ suggest that this microprotein is functional (Fig. 1d). PIGBOS has no paralogs or homologs which prevents any molecular, cellular, or physiological function from being inferred, and requires the de novo characterization of PIGBOS.

### PIGBOS is a mitochondrial outer membrane (MOM) microprotein

**Fig. 2:**
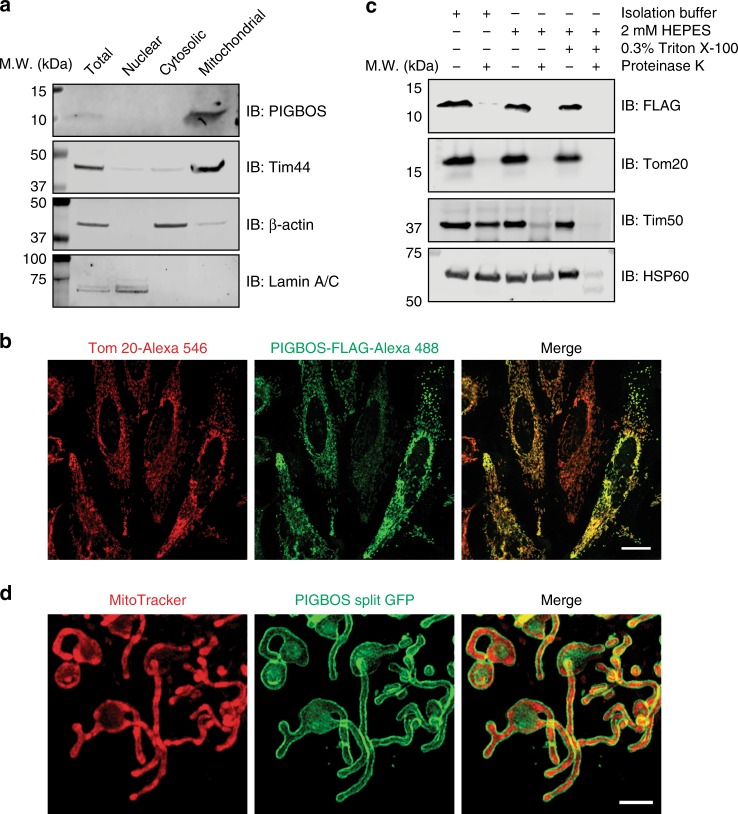
PIGBOS is localized on the mitochondrial outer membrane (MOM). **a** Western blot analysis of nuclear, cytosolic, and mitochondrial fractions from HEK293T cells identifies PIGBOS as a mitochondrial microprotein. **b** Immunofluorescence imaging of HeLa cells after transfection with PIGBOS-FLAG reveals colocalization of PIGBOS (green) with the mitochondrial marker Tom20 (red), validating PIGBOS’s mitochondrial localization. Scale bar: 20 µm. **c** Sub-mitochondrial localization using a protease protection assay reveals PIGBOS to be a mitochondrial outer membrane microprotein since proteinase K can access and degrade PIGBOS without any mitochondrial permeabilization. **d** Live cell imaging of COS-7 cells that were co-transfected with PIGBOS-3 × GFP11 and GFP (1–10) results in a green fluorescent ring around the mitochondria (MitoTracker Deep Red FM), consistent with PIGBOS localization to the MOM. Scale bar: 2.5 µm

Subcellular localization provides valuable information to assess the function of an uncharacterized protein. PIGBOS was found in the mitochondrial fraction by Western blot and proteomics, but not in any other subcellular fractions tested (Fig. 2a and Supplementary Fig. 2a). We validated PIGBOS’s mitochondrial localization by imaging exogenously expressed PIGBOS-FLAG in HeLa cells and immunofluorescence of endogenous PIGBOS in rat C6 cells (we used rat cells because of the superior performance of the rat anti-PIGBOS antibody), which showed puncta that overlap with the mitochondrial marker Tom20 (Fig. 2b and Supplementary Fig. 2b). Sequence analysis using Transmembrane Helix Prediction (TMHMM)^17^ revealed that PIGBOS is a single-pass transmembrane protein with a transmembrane region between amino acids 7–29 (Fig. 1d). To determine whether PIGBOS is localized to the inner or outer mitochondrial membrane, we used a protease protection assay^18,19^. Proteolysis of isolated mitochondria with proteinase K under conditions that retain MOM integrity led to the degradation of PIGBOS—an identical result to that of the MOM protein Tom20—indicating that PIGBOS is a MOM microprotein (Fig. 2c).

To validate this result in live cells, we turned to an optimized split GFP approach^20^ whereby three repeats of the last beta-strand of GFP (3 × GFP11) are fused to the C-terminus of PIGBOS (PIGBOS-3 × GFP11) and the non-fluorescent remainder of the GFP, i.e., GFP(1–10), is co-expressed. In this system, fluorescence is only observed if GFP11 and GFP(1–10) interact to reconstitute the intact GFP beta-barrel (i.e., GFP11 + GFP(1–10))^20^. Co-expression of PIGBOS-3 × GFP11 and the GFP(1–10) resulted in a fluorescent ring around the mitochondria (Fig. 2d and Supplementary Fig. 2c), consistent with the aforementioned biochemical data showing that PIGBOS is localized to the MOM. The same mitochondrial outline has been observed for the MOM protein Tom20^21^. Because the 3 × GFP11 must interact with cytosolic GFP(1–10) to reconstitute a fluorescent GFP, this experiment also suggested that the C-terminus of PIGBOS is cytoplasmic. Placement of 3 × GFP11 at the PIGBOS N-terminus (3 × GFP11-PIGBOS-FLAG) readily localized PIGBOS in the mitochondria but produced no fluorescence because it cannot interact with GFP(1–10) (Supplementary Fig. 2d). PIGBOS’s topology indicates that it belongs to a group of MOM proteins called signal-anchor proteins that rely on their transmembrane domains to localize the proteins to the mitochondria and anchor the proteins to the MOM^22^.

### The PIGBOS microprotein interacts with the ER protein CLCC1

**Fig. 3:**
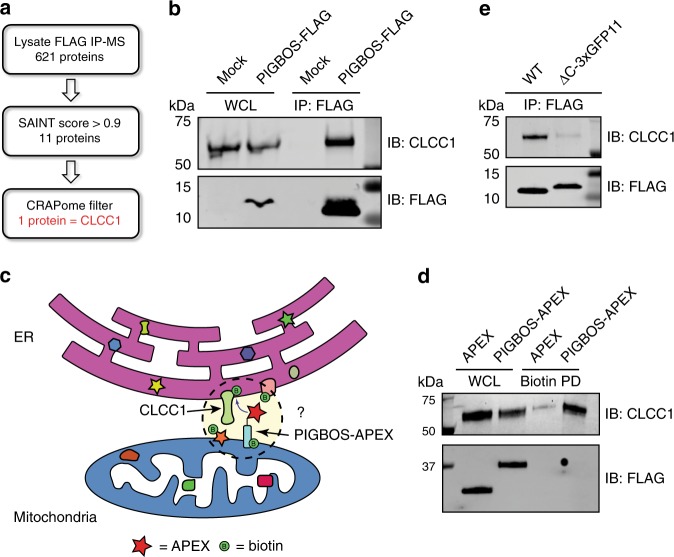
PIGBOS interacts with the ER protein CLCC1. **a** Analysis of the PIGBOS-FLAG IP-MS to remove false positives and background proteins yielded CLCC1, an ER-resident protein, as a PIGBOS interacting protein. **b** Co-immunoprecipitation of PIGBOS-FLAG enriched CLCC1 from HEK293T total cell lysates. **c** A PIGBOS-APEX construct was used to proximity label the proteome and determine whether PIGBOS and CLCC1 are near each other in living cells. **d** Enrichment of CLCC1 in PIGBOS-APEX labeling proteome indicated that it was biotinylated and, therefore, in proximity to PIGBOS-APEX. **e** Replacement of the PIGBOS C-terminal region with 3 × GFP11 (PIGBOS-ΔC-3 × GFP11-FLAG) prevented enrichment of CLCC1

Protein interaction studies can accelerate the characterization of microproteins^8,23^, so we attempted to use this strategy to characterize PIGBOS. Proteomics of immunoprecipitated PIGBOS-FLAG followed by SAINT^24^ and CRAPome^25^ analysis to remove false positives and contaminating proteins identified chloride channel CLIC-like 1 (CLCC1) as a PIGBOS-interacting protein (Fig. 3a, b and Supplementary Data 1 and Supplementary Tables 1 and 2). We validated this interaction in live cells using a proximity labeling assay^26^ with a PIGBOS-engineered ascorbate peroxidase 2 fusion protein (PIGBOS-APEX), which showed robust biotinylation and enrichment of CLCC1, whereas the ER marker Sec61b showed no enrichment and was equally biotinylated by PIGBOS-APEX and APEX control (Fig. 3c, d and Supplementary Fig. 3a). The protein interaction data indicated that PIGBOS and CLCC1 are in close proximity and that PIGBOS specifically interacts with CLCC1. A reciprocal immunoprecipitation assay with an HA-tagged CLCC1 (CLCC1-HA) enriched PIGBOS-FLAG, which further indicated the interaction between the two proteins (Supplementary Fig. 3b). CLCC1 is a putative chloride channel localized to the ER^27^, though some data suggests it might be found in the nucleus, Golgi, and plasma membrane as well^28^. Due to this ambiguity, we reassessed CLCC1 localization by imaging CLCC1-HA and the ER marker Sec61b-mCherry, which showed substantial overlap (Supplementary Fig. 4a). By contrast, counterstaining of CLCC1-HA and the Golgi marker GM130 revealed no colocalization (Supplementary Fig. 4b). Together, these experiments indicated that CLCC1 is primarily an ER protein.

### The PIGBOS C-terminus is required for the CLCC1 interaction

Based on PIGBOS’s topology, the cytosolic C-terminus of PIGBOS should mediate the interaction with CLCC1; however, testing this model by simply removing the entire PIGBOS C-terminal region (amino acids 30–54) was unsuccessful because the truncated PIGBOS(1-29)-FLAG showed no detectable expression. Instead, we swapped the 25-amino acid PIGBOS C-terminal region with the three GFP11 repeats (PIGBOS-∆C-3 × GFP11-FLAG). This PIGBOS variant has robust mitochondrial localization (Supplementary Fig. 5) but does not enrich CLCC1 after immunoprecipitation (Fig. 3e), demonstrating the requirement of the C-terminal region for an interaction with CLCC1. In an attempt to define the specific amino acids needed for binding, we mutated blocks of three consecutive amino acids of the C-terminal region to alanine (Supplementary Fig. 6a). Confocal imaging showed that all of the triple alanine PIGBOS-3 × GFP11-FLAG mutants were expressed and localized to mitochondria (Supplementary Fig. 6b). Immunoprecipitation experiments identified amino acids 30–36 of PIGBOS to be critical for CLCC1 binding since mutation of these amino acids to alanine resulted in decreased CLCC1 enrichment (Supplementary Fig. 6c). In total, these results demonstrated that the C-terminal region of PIGBOS is essential for the PIGBOS-CLCC1 interaction.

### Measuring the interaction between PIGBOS and CLCC1 in cells

**Fig. 4:**
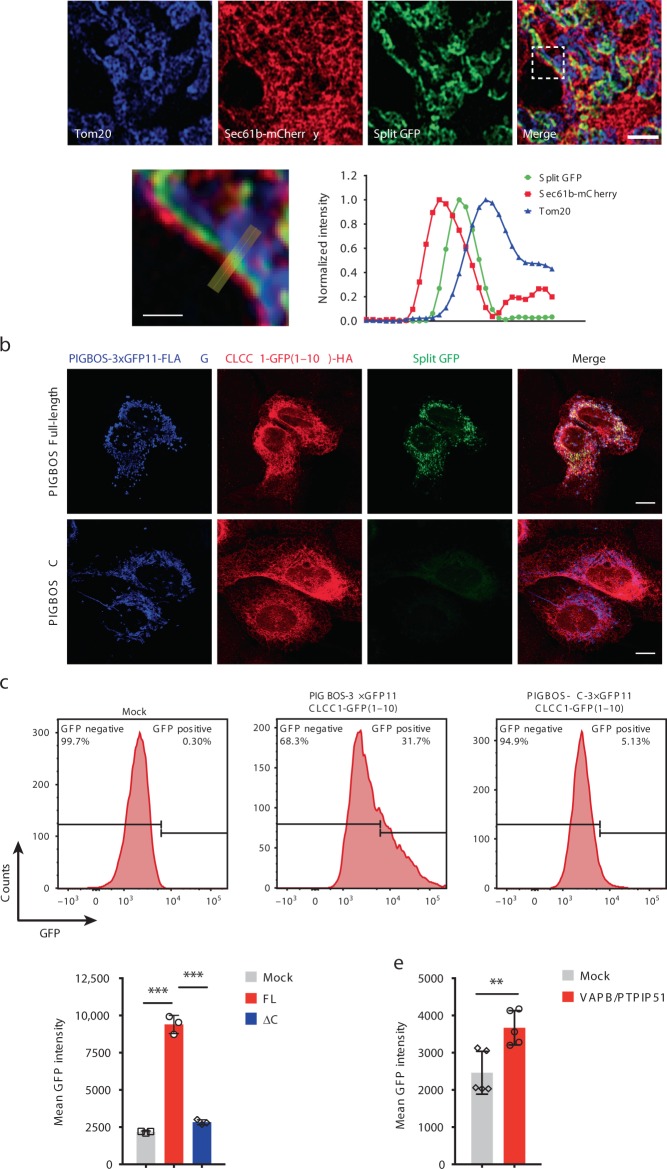
Validation of PIGBOS-CLCC1 interaction via split GFP bimolecular complementation. **a** (Top) Transfection of COS-7 cells with PIGBOS-3 × GFP11 and CLCC1-GFP(1-10) resulted in a GFP signal, which could only occur if the two proteins are close enough to interact and reconstitute a functional GFP. Scale bar: 2 µm. (Bottom) The region in the white box was enlarged, and a cross-sectional analysis of the normalized fluorescence distribution of the Tom20 (MOM), Sec61b (ER), and GFP signals places the GFP signal between the ER and MOM. Scale bar: 0.5 µm. **b** U2OS cells were co-transfected with CLCC1-GFP(1-10)-HA and PIGBOS-3 × GFP11-FLAG (or PIGBOS-ΔC-3 × GFP11-FLAG). Forty-eight hours later, cells were fixed and stained with FLAG and HA antibodies overnight before imaging. Scale bar: 10 µm. **c** Flow cytometry measurement of PIGBOS-CLCC1 interaction in HEK293T cells. HEK293T cells were co-transfected with CLCC1-GFP(1-10)-HA and PIGBOS-3 × GFP11-FLAG (or PIGBOS-ΔC-3 × GFP11-FLAG). GFP signals were assessed by flow cytometry 72 hours after transfection. **d** Quantification of mean GFP intensity in (**c**). Error bars, s.d., ***p < 0.001 (two tailed unpaired *t*-test), *n* = 3 independent experiments. **e** Flow cytometry measurement of reconstituted PIGBOS-CLCC1 split GFP intensity in HEK293T cells expressing a known ER-mitochondrial tether, VAPB/PTPIP51. Error bars, s.d., **p < 0.01 (two tailed unpaired *t*-test), *n* = 5 independent experiments. Source data for Fig. 4a, d and e are provided as a Source Data file

We next used a split-GFP bimolecular complementation strategy to image and quantify the PIGBOS-CLCC1 interaction. Co-expression of PIGBOS-3 × GFP11 and CLCC1-GFP(1-10) resulted in a robust green fluorescent signal, whereas the C-terminus truncated PIGBOS-∆C-3 × GFP11-FLAG failed to fluoresce (Fig. 4a, b). This result indicated that the reconstituted GFP fluorescence depends on PIGBOS-CLCC1 interaction, making this an ideal assay to measure the PIGBOS-CLCC1 interaction in cells. Overlap of the GFP and PIGBOS fluorescence signals indicated that not all PIGBOS is interacting with CLCC1 (Fig. 4b). In addition, spatial analysis of the fluorescence distribution revealed that the GFP signal peaks between the fluorescent reporter signals for the MOM (Tom20) and ER (Sec61b-mCherry), providing further evidence that the PIGBOS-CLCC1 interaction occurs at the ER-mitochondria interface (Fig. 4a).

To assess the PIGBOS-CLCC1 interaction in bulk and in live cells, we developed a flow cytometry experiment to measure the amount of reconstituted GFP^29^. Cells expressing PIGBOS-3 × GFP11 and CLCC1-GFP(1–10) had a robust GFP signal with ~30% of the cellular population above the minimum threshold and significantly higher mean GFP intensity compared to mock transfected controls. By contrast, the C-terminus truncated PIGBOS-∆C-3 × GFP11 showed no detectable GFP signal, supporting that the C-terminus of PIGBOS is necessary for its interaction with CLCC1 (Fig. 4c, d and Supplementary Fig. 7).

### The PIGBOS-CLCC1 interaction is not a tether

Given that CLCC1 and PIGBOS are ER and mitochondria transmembrane proteins, respectively, as well as the observation that PIGBOS-3 × GFP11 and CLCC1-GFP(1-10) generated the GFP fluorescence right between ER and mitochondria (Fig. 4a), we considered that they interact at ER-mitochondria contact sites. This model requires CLCC1 to be present in the portion of the ER that contacts the mitochondria which is referred to as the mitochondria-associated ER membrane (MAM). The MAM is an important inter-organelle junction in the cell that mediates cellular calcium levels, lipid metabolism, mitochondrial dynamics, and apoptosis^30,31^. We isolated the MAM using standard fractionation protocols^32,33^ and detected CLCC1 by Western blot along with the calnexin, a known MAM protein (Supplementary Fig. 8a), which, consistent with our assumption, indicated that CLCC1 is in the ER fraction with close proximity to mitochondria.

**Fig. 5:**
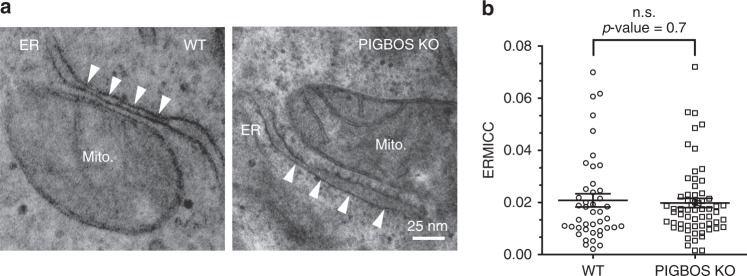
PIGBOS shows no effect on modulation of ER-mitochondria contacts. **a** Representative transmission electron microscopy images from WT and PIGBOS-KO U2OS cells showed no remarkable differences in ER-mitochondria contacts. ER and mitochondria contact sites are indicated by white arrows. **b** Quantitation of the normalized ER-mitochondria contact coefficient (ERMICC) did not identify a significant difference between ERMICC of WT vs. PIGBOS-KO U2OS cells. Data are collected from two independent experiments and pooled from 43 WT mitochondria (eight cells), and 62 PIGBOS-KO mitochondria (seven cells) and the bar graph is the ERMICC ± s.e.m. with the *p*-value calculated using two tailed unpaired *t*-test with all data points included in the calculation. Source data for Fig. 5b are provided as a Source Data file

There are only a handful of reported protein-protein interactions between the ER and mitochondria in mammalian cells, and most of these interactions are implicated in ER-mitochondria tethering^26,33,34^. For example, a known ER-mitochondria tether is comprised of the vesicle-associated membrane protein-associated protein B/C (VAPB) in the ER and protein tyrosine phosphatase-interacting protein 51 (PTPIP51) in the mitochondria. Overexpression of VAPB and PTPIP51 increases the number of ER-mitochondria contact sites^35^. To test a role for PIGBOS and CLCC1 in ER-mitochondria tethering, we used transmission electron microscopy to visualize ER-mitochondria contacts in WT and PIGBOS-KO U2OS cells (Fig. 5 and Supplementary Fig. 9). We observed no changes to ER-mitochondria contact sites (Fig. 5a), and quantitation of the normalized ER-mitochondria contact coefficient (ERMICC)^36^ revealed no significant differences in ER-mitochondria contacts in WT versus PIGBOS-KO cells (Fig. 5b). Consistent with the result that PIGBOS-CLCC1 is not a tether, overexpression of the known ER-mitochondria tether VAPB and PTPIP51 resulted in a change in ER-mitochondria morphology, in which the ER was localized near to mitochondria, whereas overexpression of PIGBOS and CLCC1 did not (Supplementary Fig. 10). In addition, the levels of CLCC1 in the MAM fraction from PIGBOS-KO and WT HEK293 cells are equivalent, which indicated that the amount of CLCC1 in the MAM does not depend on PIGBOS (Supplementary Figs. 8b and 11).

Since the PIGBOS-CLCC1 interaction does not influence the ER-mitochondria contacts, we asked whether cells with changes to the number of ER-mitochondria contacts can regulate the interaction between PIGBOS and CLCC1. We tested this idea by measuring PIGBOS-CLCC1 split GFP intensity in cells expressing VAPB and PTPIP51. Using our quantitative flow cytometry assay, we found that the overexpression of VAPB and PTPIP51 significantly increased interactions between PIGBOS and CLCC1 as indicated by greater number of GFP positive cells and mean GFP intensity (Fig. 4e and Supplementary Fig. 12a), while total concentrations of PIGBOS and CLCC1 were unchanged (Supplementary Fig. 12b). This result demonstrated that the PIGBOS-CLCC1 interaction can be regulated by modulation of ER-mitochondria contacts, and further bolstered the model of CLCC1 and PIGBOS interacting at ER-mitochondria contact sites.

### PIGBOS modulates UPR in the ER

A genetics study in mice identified lower CLCC1 expression levels as the driver of a neurodegenerative phenotype, with mechanistic studies supporting increased UPR as the underlying cause^27^. We reproduced this finding and observed that treatment of CLCC1 knockdown (KD) cells with tunicamycin (TM), an inducer of ER stress, led to increased XBP1 splicing, an established marker for UPR (Supplementary Fig. 13a). Since PIGBOS interacts with CLCC1, we hypothesized that PIGBOS may have a role in UPR signaling, which, if true, would provide the first example of a mitochondrial protein regulator of UPR.

**Fig. 6:**
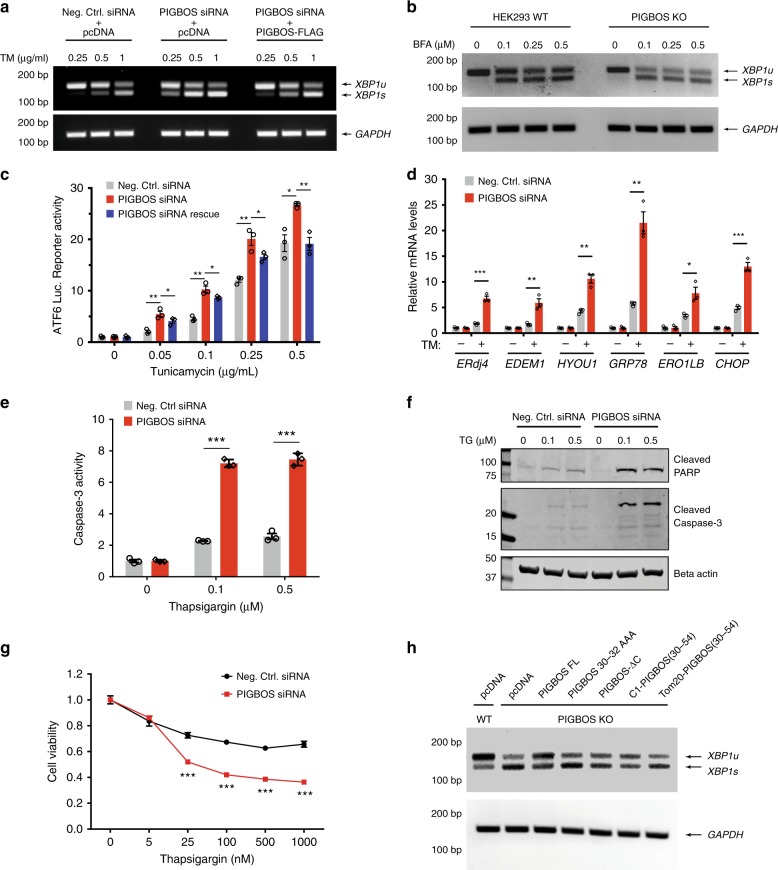
PIGBOS regulates the amplitude of UPR, and apoptosis. **a** PIGBOS-KD and control HEK293 cells were treated with indicated concentrations of tunicamycin (TM) followed by RT-PCR analysis of XBP-1 splicing (unspliced XBP1 (XBP1u) and spliced XBP1 (XBP1s)). GAPDH was used as a loading control. A stronger UPR correlate with higher XBP1s/XBP1u ratio in PIGBOS-KD cells, which could be rescued by expression of a siRNA-resistant PIGBOS-FLAG. **b** XBP-1 mRNA splicing was measured in PIGBOS-KO and control HEK293 cells treated with indicated doses of brefeldin A (BFA) for 3 h. **c** ATF6-dependent luciferase reporter measures the activation of another branch of the UPR pathway. PIGBOS-KD led to increased luciferase activity indicative of a greater UPR, and the expression of the siRNA-resistant PIGBOS-FLAG reversed this effect. **d** RT-qPCR quantitation of a panel of UPR target genes in PIGBOS-KD and control HEK293 cells after an 8-hour treatment with vehicle or 1 μg/ml of TM. **e** Caspase-3 activity in mock and PIGBOS-KD U2OS cells treated with thapsigargin (TG) for 27 h. **f** Cleaved PARP and caspase-3 levels were measured by Western blot in PIGBOS-KD and control U2OS cells treated with TG for 27 h. **g** PIGBOS-KD and control U2OS cells were treated with indicated doses of TG for 48 h followed by cell viability measurements using MTT. **h** HEK293 PIGBOS-KO and WT cells were transfected with PIGBOS variants constructs as indicated. Forty-eight hours later, cells were incubated with 1 µg/ml of tunicamycin for 3 h. XBP1 splicing activity was measured by RT-PCR. Error bars, s.e.m. The *p*-values were calculated using two tailed unpaired *t*-test. *p < 0.05, **p < 0.01, ***p < 0.001, *n* = 3 experiments. Source data for Fig. 6c–e and 6g are provided as a Source Data file

We tested whether PIGBOS regulates UPR by treating PIGBOS siRNA KD or CRISPR-Cas9 knockout (KO) cells (Supplementary Figs 11 and 13b) with TM. In the absence of PIGBOS, we observed an increased sensitivity of cells to TM—a stronger UPR at lower TM concentrations—detected as elevated levels of spliced XBP1 and an increased ratio of spliced XBP1 to unspliced XBP1 (XBP1s/XBP1u) (Fig. 6a and Supplementary Fig. 13c–e). Furthermore, expression of an siRNA resistant PIGBOS-FLAG construct in the PIGBOS-KD cells partially reversed the phenotype by normalizing the sensitivity of UPR to TM (Fig. 6a), verifying that PIGBOS is responsible for the observed effects on UPR. Overexpression of PIGBOS in WT cells resulted in the desensitization of cells to UPR with decreased XBP1 splicing (Supplementary Fig. 13f), providing additional evidence for a specific role for PIGBOS in UPR. Taken together, these results suggested that modulating PIGBOS levels can in turn modulate cellular sensitivity towards ER stress.

To assess the generality of PIGBOS regulation of UPR, we tested different ER stressors and measured the activity of the ATF6 branch of the UPR pathway. Loss of PIGBOS also showed heightened sensitivity to thapsigargin (TG) and Brefeldin A (BFA), two mechanistically distinct UPR activators, indicating that PIGBOS is downstream of both types of UPR induction (Fig. 6b and Supplementary Fig. 13g). Analysis of the ATF6 branch of UPR in WT and PIGBOS-KD cells using an ATF6 luciferase reporter assay^37^ revealed increased ATF6-driven luciferase activity in PIGBOS-KD cells, which was rescued by the expression of siRNA resistant PIGBOS-FLAG (Fig. 6c). To obtain the most comprehensive view of PIGBOS in its regulation of UPR, we measured mRNA levels of a panel of UPR-regulated genes that promote degradation of misfolded proteins (*ERdj4* and *EDEM1*), protein folding (*HYOU1*, *GRP78*, and *ERO1LB*), and apoptosis (*CHOP*). Upon UPR induction with TM, the loss of PIGBOS led to dramatic increases in the levels of all UPR target genes measured, indicating increased UPR signaling across all the branches (IRE1, PERK, and ATF6) (Fig. 6d and Supplementary Fig. 14a). Meanwhile, PIGBOS overexpressing cells showed the opposite effect, in which the UPR target genes showed less UPR activation, indicating a tunable modulation of ER stress by PIGBOS microprotein levels (Supplementary Fig. 14b). We then confirmed via Western blot that TM treatment of PIGBOS-KD cells led to higher ATF4 and CHOP protein levels (Supplementary Fig. 13h). These data identified PIGBOS as a heretofore unknown mitochondrial regulator of UPR, and the only known microprotein linked to the regulation of cell stress or inter-organelle signaling.

We then assessed whether the PIGBOS-CLCC1 interaction is regulated by ER stress. Immunoprecipitation of PIGBOS-FLAG in TM or TG treated cells showed similar amounts of co-eluted CLCC1, suggesting ER stress has no effect on the PIGBOS-CLCC1 interaction (Supplementary Fig. 15a). To confirm this result, we used flow cytometry analysis to evaluate PIGBOS-CLCC1 split GFP intensity in TM or TG treated cells. No significant differences with regard to either GFP positive cells or mean GFP intensity were observed in cells with ER stress up to 7 h (Supplementary Fig. 15b–d).

UPR is a dedicated signaling network to deal with unfolded protein stress in the ER. Recent studies revealed emerging evidence of a cellular response to unfolded protein accumulation in the mitochondria, referred to as MitoUPR, which is induced by distinct stimuli and results in different response mechanisms with some overlapping targeting genes compared to the canonical ER UPR^38,39^. To test whether PIGBOS has a role in MitoUPR, we used bardoxolone (CDDO) to chemically induce mitochondrial protein misfolding in PIGBOS-KO and WT cells, and evaluated the MitoUPR targeting gene expression (i.e., *HSPD1* and *CHOP*)^40,41^. Both genes increased dramatically upon CDDO treatment, however, there was no notable difference between PIGBOS-KO and WT cells (Supplementary Fig. 16). It is worth noting that *CHOP* level was significantly increased in PIGBOS-KD cells compared to WT cells in response to TM induced ER stress, indicating that PIGBOS specifically regulates ER UPR.

### PIGBOS regulates ER stress-induced apoptosis

Previous studies demonstrated that prolonged ER stress would lead to apoptosis if cells fail to cope with accumulating misfolded proteins^9^. Since cells lacking PIGBOS are more sensitive to UPR, we predicted that these cells would undergo apoptosis more readily. Indeed, we observed increased apoptosis in PIGBOS-KD and PIGBOS-KO cells treated with TG or TM using a caspase-3 and PARP-cleavage assay (Fig. 6e, f, and Supplementary Fig. 17a–c). PIGBOS-KD cells were also less viable than control cells during TG-induced cell stress (Fig. 6g). Interestingly, treating cells with staurosporine (STS), a non-selective protein kinase inhibitor and apoptosis inducer, revealed neglectable differences of cell viability in PIGBOS-KD and WT cells, which indicated a specific connection between ER stress and PIGBOS regulation (Supplementary Fig. 17d). Together, these results showed that loss of PIGBOS increases cellular sensitivity to ER stress, which in turn increases apoptosis and links PIGBOS levels to the ability of cells to survive stress.

### PIGBOS-CLCC1 interaction is necessary for PIGBOS function

To confirm that the increased UPR sensitivity in PIGBOS diminished cells is mediated by PIGBOS-CLCC1 interaction, we performed rescue experiments with non-CLCC1 binding PIGBOS mutants. Only full-length PIGBOS microprotein reversed the XBP1 splicing phenotype, whereas the non-CLCC1-binding PIGBOS mutants with the C-terminus truncation (PIGBOS-∆C-3 × GFP11-FLAG) or the triple alanine mutant of aa 30–32 (PIGBOS-3 × GFP11-FLAG, 30–32 AAA) showed similar activity as in cells lacking PIGBOS (Fig. 6h and Supplementary Fig. 18a). Furthermore, expression of PIGBOS full-length protein, but not C-terminus truncated PIGBOS (PIGBOS-∆C-3 × GFP11-FLAG), in PIGBOS-KD cells is able to partially rescue the ER stress triggered apoptosis (Supplementary Fig. 18b). In addition, we made chimeric PIGBOS variants to anchor the PIGBOS cytosolic region (i.e., aa 30–54) to either the ER membrane (C1-PIGBOS(30–54)) or MOM (Tom20-PIGBOS(30–54)). Confocal images demonstrated accurate subcellular localization (Supplementary Fig. 18c and d), but they were unable to interact with CLCC1 efficiently (Supplementary Fig. 18e). In addition, Tom20-PIGBOS(30–54) failed to rescue XBP1 splicing activity, while C1-PIGBOS(30–54) showed decreased XBP1 splicing activity but this might be due to C1 peptide having independent activity at the ER^42^ (Fig. 6h and Supplementary Fig. 18a).

The Tom20-PIGBOS(30–54) experiment demonstrates that placing the PIGBOS C-terminal region at MOM is insufficient for mediating the PIGBOS-CLCC1 interaction and regulating the ER stress response. As a small protein, we suspect that the different parts of PIGBOS are not easily separated from each other into functional domains, which is why we refer to them as regions. Unlike large, multidomain proteins the separation and swapping of the C-terminal region may affect the secondary or tertiary structure of PIGBOS, and in the process inhibit its ability to bind CLCC1 or partake in UPR signaling. PIGBOS belongs to a class of single-pass transmembrane proteins in which the transmembrane regions (oligomerization state, tilt angle and topology) are essential to control structure and function. TMDOCK analysis predicts that the PIGBOS transmembrane region is a homodimer (Supplementary Fig. 19a), providing evidence for PIGBOS having a tertiary structure^43^. To test this prediction, we transfected cells with human PIGBOS-FLAG and human PIGBOS-HA, or human PIGBOS-FLAG and rat PIGBOS-HA and performed FLAG immunoprecipitations. We found human PIGBOS-FLAG interacts with human and rat PIGBOS-HA providing experimental evidence for a PIGBOS oligomerization and a tertiary structure for this microprotein (Supplementary Fig. 19b, c). PIGBOS might make up for its diminutive size by oligomerizing into a tertiary structure that can provide a highly specific and unique protein structure necessary for CLCC1 binding. Future structure studies of PIGBOS and PIGBOS-CLCC1 complex will elucidate this structure in greater detail and define the role of PIGBOS tertiary structure in PIGBOS function.

## Discussion

Understanding how cells respond to stress is of importance. These pathways are required for maintaining homeostasis and cell health, and their dysregulation can lead to disease. For example, UPR dysfunction contributes to accumulation of key disease-related proteins, and thus plays an essential role in the pathogenesis of many neurodegenerative disorders, including Alzheimer’s disease, Parkinson’s disease, and Huntington’s disease^14,15^. Cells with an insufficient capacity to handle protein production begin to accumulate unfolded or misfolded proteins, which causes ER stress and triggers UPR. Elegant genetic studies in yeast revealed the conserved ER machinery that is activated during UPR and mediates the signaling pathways needed to express the genes necessary to cope with stress. The essential proteins in the eukaryotic ER stress response machinery include the kinases PERK and IRE1, and the proteolytically activated transcription factor ATF6. All three of these foundational genes are localized to the ER, identifying the ER as the hub for regulating UPR.

The ER stress response consists of intricate signaling networks across the entire cell with spatial and temporal regulation that requires communications between ER and other intracellular organelles. In particular, mitochondria play a vital role in crosstalk with ER during UPR by providing energy for protein folding in the ER as well as activating apoptosis if the stress remains unmitigated. However, the communication between the ER and mitochondria in the context of UPR is elusive. One way to solve how ER-originating cellular signaling pathways such as UPR are propagated in the cell is to identify regulators of inter-organelle communication at contact sites. The list of known ER-mitochondria contact proteins in mammals is relatively short^34^, and only one of these proteins, the mitochondrial fusion protein Mfn2, has been implicated in UPR^44^. The role of Mfn2 is confounding because it is both an ER and mitochondrial resident protein and it is a tether that regulates ER-mitochondria contact sites^36,45^, which makes it difficult to dissect whether Mfn2 directly controls UPR or whether another protein complex at ER-mitochondria contact sites might be responsible and is coincidentally disrupted by the loss of Mfn2. The microprotein PIGBOS is the latest member to join the short list of functional protein complexes at the ER-mitochondria interface but is the only mitochondria-specific protein to date to modulate UPR in the ER.

PIGBOS has a unique genomic localization, which is on the opposite strand of the PIGB gene. In human, the first exons of these two genes share an overlapping region, raising the question as to whether expression of one gene can influence the other, or whether the two genes are co-regulated. We knocked down *PIGBOS* and *PIGB* respectively in HEK293 cells using siRNAs, and measured gene expression levels of the other gene. We found that KD of one gene does not interfere with the expression of the other one (Supplementary Fig. 20a, b). In addition, we demonstrated that *PIGBOS* mRNA levels decreased in LPS treated RAW 264.7 cells. Interestingly, *PIGB* mRNA also decreased to a similar extent in the same cells (Supplementary Fig. 20c). These results implied that PIGBOS and PIGB might be co-regulated by the same promoter and transcription factors. Future work will be performed to fully address this question and investigate whether it can be applied to other antisense microproteins.

The characterization of microproteins has led to unprecedented mechanistic insights in other pathways, such as the role of minion in muscle fiber formation^7^ and CYREN in the cell-cycle dependent inhibition of non-homologous end joining (NHEJ) repair^8^. Functional microproteins, including PIGBOS, demonstrate that they are as vital to cellular and physiological functions as any other protein; however, unlike large multidomain proteins, microproteins present challenges in trying to understand their structure-function relationship. For example, due to the small size, normal protein immunoprecipitation experiments might not be able to enrich the binding partners because microproteins may not have enough surface area to bind an antibody while maintaining a microprotein-protein interaction partner^46^. As a result, immunoprecipitation with overexpressed epitope-tagged microproteins is the most reasonable way to characterize the interactomes of microproteins and has successfully been used to find several bona fide microprotein–protein interactions^1,8,23^. Furthermore, microprotein regions, as opposed to classical protein domains, cannot be swapped and retain their functions as demonstrated by TOM20-PIGBOS(35–54) fusion which loses PIGBOS’s binding and biological activity.

The functional assignment of PIGBOS in this study has revealed the ability of a mitochondrial protein to regulate UPR in the ER, which makes PIGBOS unique and demonstrates the existence of non-ER proteins in the regulation of UPR. Our work also reveals that inter-organelle interactions can be mediated by microproteins and raises the possibility that other inter-organelle or inter-cellular protein interactions at membrane contact sites might involve microproteins. Furthermore, recent work has demonstrated that the dysregulation of the ER stress response is implicated in human disease including viral infections, neurodegeneration, cancer, and diabetes^47^. Given the importance of UPR in biology and disease, future studies on PIGBOS’s role in UPR should afford additional insights and may provide methods for regulating this pathway for therapeutic applications.

## Materials and methods

### Materials

Cell lines used in the study were purchased from ATCC, HEK293 (CRL-1573), HEK293T (CRL-11268), U2OS (HTB-96), HeLa (CCL-2), and COS-7 (CRL-1651). siRNAs used in this study were purchased from GE Healthcare Dharmacon, Inc. and listed in Supplementary Table 3. Sequences of RT-qPCR primers are listed in Supplementary Table 4. Sequences of gRNA protospacers and genotyping primers for PIGBOS knockout are shown in Supplementary Table 5. Information of antibodies used in this study are shown in Supplementary Table 6. DNA constructs and corresponding subcloning primers are listed in Supplementary Tables 7 and 8 respectively.

### Animal care

All animal procedures were approved by the Institutional Animal Care and Use Committee of the Salk Institute and were conducted in accordance with the PHS Policy on Humane Care and Use of Laboratory Animals (PHS Policy, 2015), the U.S. Government Principles for Utilization and Care of Vertebrate Animals Used in Testing, Research and Training, the NRC Guide for Care and Use of Laboratory Animals (8th edition) and the USDA Animal Welfare Act and Regulations. All animals were housed in an AAALAC accredited facility in a climate-controlled environment (65–72 degrees Fahrenheit, 30–70% humidity) under 12-h light/12-h dark cycles. Upon arrival, animals were physically examined by veterinary staff for good health and acclimated for at least two weeks prior to initiation of antiserum production. Each animal was monitored daily by the veterinary staff for signs of complications and weighed every two weeks. Routine physical exams were also performed by the veterinarian quarterly on all rabbits and guinea pigs. For the production of antiserum against human PIGBOS, three 10 to 12-week old, female New Zealand white rabbits, weighing 3.0–3.2 kg at beginning of the study, were procured from Irish Farms (I.F.P.S. Inc., Norco, California, USA). Rabbits were provided with ad libitum feed (5326 Lab Diet High Fiber), micro-filtered water and weekly fruits, vegetables and alfalfa hay for enrichment. For the production of rat PIGBOS antiserum in guinea pigs, four 10–12-week old, female Hartley guinea pigs, weighing 700–750 g at the beginning of the study, were procured from Charles River Laboratories. Guinea pigs were provided with ad libitum feed (5025 Lab Diet), micro-filtered water and weekly fruits and vegetables for enrichment.

### Preparation of antigens

Peptides were synthesized by RS Synthesis (Louisville, KY), HPLC purified to >95%, and amino acid sequence verified by mass spectrometry. Peptides were conjugated to maleimide activated Keyhole Limpet Hemocyanin (KLH) per manufacturer’s instructions (ThermoFisher, Waltham MA). Specific peptides used to generate antisera were as follows: Cys32 human PIGBOS(32-42)-NH_2_, CAKDQKELKEK- NH_2_; Cys32 rat PIGBOS (32–54), CSRDQKELKELVKILQESEEKRS.

### Injection and bleeding of animals

The antigen was delivered to host animals using multiple intradermal injections of the peptide-KLH conjugate in Complete Freund’s Adjuvant (initial inoculation) or incomplete Freund’s adjuvant (booster inoculations) every three weeks for rabbits and once every four weeks for guinea pigs. Animals were bled, <10% total blood volume, one week (rabbits) or two weeks (guinea pigs) following booster injections and bleeds screened for titer and specificity. Rabbits were administered 1–2 mg/kg Acepromazine IM prior to injections of antigen or blood withdrawal. Guinea pigs were anesthetized using inhalation isoflurane maintained at 2–2.5% prior to injections and bleedings. At the termination of the study, rabbits were exsanguinated under anesthesia (ketamine 50 mg/kg and acepromazine 1 mg/kg, IM) and euthanized with an overdose of pentobarbital sodium and phenytoin sodium (1 ml/4.5 kg of body weight IC to effect). Guinea pigs were exsanguinated via cardiac puncture under inhalation anesthesia (isoflurane maintained at 2–2.5%). After blood was collected death of animals was confirmed. All animal procedures were conducted by experienced veterinary technicians, under the supervision of Salk Institute veterinarians.

### Characterization and purification of antisera

Each bleed from each animal was tested at multiple doses for the ability to recognize the synthetic peptide antigen; bleeds with highest titers were further analyzed by western immunoblot for the ability to recognize the full-length endogenous protein and to check for cross-reactivity to other proteins. Antisera with the best characteristics of titer against the synthetic peptide antigen, ability to recognize the endogenous protein, and specificity were antigen affinity purified and used for all studies. Rabbit PBL#7410 anti-human PIGBOS and guinea pig PBL#114 anti-rat PIGBOS were purified using human PIGBOS(32–54) coupled to Affi-Gel 10 (Bio-Rad Laboratories, Hercules CA) or Cys32 rat PIGBOS (32–54) covalently attached to Sulfolink agarose (ThermoFisher, Waltham MA), respectively. Coupling of peptides to resins was per manufacturer’s instructions. To ensure that the same batch of purified antibodies could be used for this and future studies, large volumes, ~20 ml sera, from bleeds with similar profiles were purified.

### MS sample preparation and instrumentation

Samples were precipitated with trichloroacetic acid (TCA, MP Biomedicals, #196057) overnight at 4 °C. Dried pellets were dissolved in 8 M urea, reduced with 5 mM tris(2-carboxyethyl)phosphine hydrochloride (TCEP, Thermo, #20491) and alkylated with 10 mM iodoacetamide (Sigma, I1149). Proteins were then digested overnight at 37 °C with trypsin (Promega, V5111). The reaction was quenched with formic acid at a final concentration of 5% (v/v). The digested samples were analyzed on a Q Exactive mass spectrometer (Thermo). The digest was injected directly onto a 30 cm, 75 µm ID column packed with BEH 1.7 µm C18 resin (Waters). Samples were separated at a flow rate of 200 nl/min on a nLC 1000 (Thermo). Buffer A and B were 0.1% formic acid in water and acetonitrile, respectively. A gradient of 5–40% B over 110 min, an increase to 50% B over 10 min, an increase to 90% B over another 10 min and held at 90% B for a final 10 min of washing was used for 140 min total run time. The column was re-equilibrated with 20 µl of buffer A prior to the injection of sample. Peptides were eluted directly from the tip of the column and nanosprayed directly into the mass spectrometer by application of 2.5 kV voltage at the back of the column. The Q Exactive was operated in a data-dependent mode. Full MS1 scans were collected in the Orbitrap at 70 K resolution with a mass range of 400–1800 *m/z* and an AGC target of 5e^6^. The ten most abundant ions per scan were selected for MS/MS analysis with HCD fragmentation of 25NCE, an AGC target of 5e^6^ and minimum intensity of 4e^3^. Maximum fill times were set to 60 ms and 120 ms for MS and MS/MS scans respectively. Quadrupole isolation of 2.0 *m/z* was used, dynamic exclusion was set to 15 s and unassigned charge states were excluded. Protein and peptide identification were done with Integrated Proteomics Pipeline—IP2 (Integrated Proteomics Applications). Tandem mass spectra were extracted from raw files using RawConverter^48^ and searched with ProLuCID^49^ against human UniProt database appended with microprotein sequences. The search space included all fully-tryptic and half-tryptic peptide candidates with a maximum of two missed cleavages. Carbamidomethylation of cysteine was counted as a static modification. Data was searched with 50 ppm precursor ion tolerance and 50 ppm fragment ion tolerance. Data was filtered to 10 ppm precursor ion tolerance post search. Identified proteins were filtered using DTASelect^50^ and utilizing a target-decoy database search strategy to control the false discovery rate to 1% at the protein level.

### Extraction of rat tissues for Western blot analysis

Tissues were extracted using a mixture of hot (90 °C) 1 N acetic acid/0.1 N HCl, homogenized with a Polytron blender, centrifuged at 30,000 × *g* for 30 min at 4 °C, and supernatants removed and filtered through 5 μm syringe filters. Supernatants were enriched for microproteins as described^51^, except that Bond Elut C18 cartridges were used.

### Confocal imaging

For fixed cell imaging, cells were seeded onto coverslips (Fisher Scientific, 12-541-B) pre-treated with 50 µg/mL poly-L-lysine (Sigma, P1399). The next day, cells were transfected with constructs as indicated using Lipofectamine 2000. Tweny-four or forty-eight hours post-transfection, cells were fixed with 4% paraformaldehyde (Polysciences, Inc., #18814) and permeabilized with fresh 0.1% saponin (Alfa Aesar, A18820). After incubating with 4% BSA in PBS for 1 hour at room temperature, cells were stained with corresponding primary antibodies overnight at 4 °C. Then the cells were washed three times with PBS, followed by incubating with Alexa Fluor-labeled secondary antibodies for 1 hour at room temperature. If necessary, nuclei were counterstained with Hoechst 33258 (Sigma, #94403, 1:2000 in PBS). After three PBS washes, the coverslip was mounted on slides using Prolong® Gold Antifade Mountant (Life Technologies, P36930). For live cell imaging, COS-7 cells were seeded onto 4-well chambered cover glass (Cellvis, C4-1.5H-N), which was pre-treated with 50 µg/mL poly-L-lysine (Sigma, P1399). The next day, cells were transfected with constructs as indicated using Lipofectamine 2000. Twenty-four hours post-transfection, cells were treated with MitoTracker Deep Red FM (Life Technologies, M22426) to label mitochondria. Cell culture medium was then changed to phenol-red free DMEM + 10% FBS and imaged at 37 °C and 5% CO_2_. All samples were imaged using a Zeiss LSM 880 Airyscan confocal microscope with a 63 × 1.4NA oil immersion objective at 2 × Nyquist pixel and z-stack step sizes, then processed using automatic filter settings in Zen Black (Zeiss) software. Images were then analyzed using FIJI software.

### Subcellular fractionation (including MAM)

Subcellular fractionation of nuclei, mitochondria, ER, MAM and cytosol from HEK293T cells was performed following the previously described protocols^32,33^. Cells were homogenized in isolation buffer (225 mM mannitol, 75 mM sucrose, 0.1 mM EGTA, 30 mM Tris-HCl pH 7.4) until 90% of cells were broken. Then, the homogenate was centrifuged at 600 × *g* for 10 min three times to clarify the supernatant. The pellet (nuclear fraction) was washed three times with isolation buffer and resuspended in RIPA buffer. Collected supernatant was centrifuged for 15 min at 7000 × *g* for obtaining crude mitochondria. The crude mitochondria were washed with isolation buffer, and 10% were resuspended in RIPA buffer, the other 90% were used to isolate pure mitochondria and MAM (see below). The collected supernatant was centrifuged at 20,000 × *g* for 30 min to remove the plasma membrane. Then, the supernatant was centrifuged at 100,000 × *g* for 1 h and the pellet was resuspended for the ER fraction and the supernatant was kept for the cytosolic fraction. For pure mitochondria and MAM fraction, the crude mitochondria pellet was resuspended in 2 mL MRB buffer (250 mM mannitol, 5 mM HEPES pH 7.4, 0.5 mM EGTA), and the fraction was added on the top of 30% percoll medium (225 mM mannitol, 25 mM HEPES pH 7.4, 1 mM EGTA, 30% percoll (v/v)) in an ultracentrifuge tube. Centrifugation was performed at 95,000 × *g* for 30 min. The bottom layer band was diluted with 10 volumes of MRB buffer and centrifuged at 6300 × *g* for 15 min twice, the pellet was then saved as pure mitochondria. The upper layer band was diluted with 10 volumes of MRB buffer and centrifuged at 6300 × *g* for 15 min, the supernatant was collected and centrifuged again at 100,000 × *g* for 1 h and the pellet was collected as MAM fraction.

### Proteinase K protection assay

Proteinase K protection assay was performed as previously described^18^. Briefly, mitochondria were isolated from HEK293T cells expressing PIGBOS-FLAG and then equally divided into six samples. Samples were resuspended and incubated on ice for 30 min in isolation buffer (225 mM mannitol, 75 mM sucrose, 50 mM HEPES pH 7.5), 2 mM HEPES (pH 7.5) or 2 mM HEPES + 0.3 % (v/v) Triton X-100 (two samples for each condition). Then samples were treated with 0.5 μL of proteinase K (New England Biolabs P8107S) on ice for 30 min (one sample for each condition). The reaction was inactivated by adding PMSF to a final concentration of 1 mM. The resulting samples were precipitated with 30% (v/v) TCA, and the pellet was washed with cold acetone and resuspended in SDS loading buffer. Protein levels were visualized by Western blotting using indicated antibodies.

### APEX labeling in live cells

Biotin-phenol labeling in live cells was performed as previously described^52^. Briefly, PIGBOS-APEX fusion proteins or APEX control were transiently transfected into HEK293T cells using Lipofectamine 2000. Twenty-four-hours post-transfection, cell culture medium was changed to fresh growth medium containing 500 μM biotin-tyramide (CDX-B0270, Adipogen). After 30 min incubation at 37 °C, H_2_O_2_ was added to each plate at a final concentration of 1 mM and the plates were gently agitated for 1 min. Cells were then washed three times with quenching solution (5 mM Trolox, 10 mM sodium azide and 10 mM sodium ascorbate in PBS) and the pellet was collected by centrifugation at 1000 × *g* for 5 min.

### Immunoprecipitations

Constructs as indicated and corresponding controls were transfected into a 10-cm dish of HEK293T cells using Lipofectamine 2000 according to manufacturer’s protocol. Forty-eight-hours post-transfection, cells were harvested and lysed in lysis buffer (50 mM Tris pH 7.4, 150 mM NaCl, 1% Triton X-100) supplemented with Roche complete protease inhibitor cocktail tablet and 1 mM PMSF. Cells were lysed on ice for 20 min followed by centrifugation at 10,000 × *g* for 10 min at 4 °C to remove cell debris. Cell lysates were added to pre-washed mouse IgG agarose beads (Sigma A0919) and rotated at 4 °C for 1 h. The supernatants were collected and added to pre-washed anti-FLAG M2 Affinity Gel (Sigma, A2220) or anti-HA agarose beads (Sigma, A2095). The suspensions were rotated at 4 °C overnight and washed four times with 1 × TBST. Bound proteins were eluted with 3 × FLAG peptide (Sigma, F4799) or HA peptide (Sigma, I2149) at 4 °C for 1 h. In some experiments, bound proteins were eluted by adding SDS loading buffer and boiled at 95 °C for 10 min. The eluents were then separated by SDS-PAGE and analyzed by Western blotting using indicated antibodies.

### Flow cytometry analysis of PIGBOS-CLCC1 interaction

HEK293T cells were co-transfected with CLCC1-GFP(1-10)-HA and PIGBOS-3 × GFP11-FLAG (or PIGBOS-ΔC-3 × GFP11-FLAG). Seventy-two hours after transfection, cells were washed once by PBS and resuspended in FACS buffer (PBS + 0.3% BSA) before analyzed by BD FACSCanto II system. To measure PIGBOS-CLCC1 interaction during ER stress, 2 µg/ml of TM (Tocris Bioscience, #3516) or 400 nM of TG (Tocris Bioscience, #1138) was added to cell culture media as indicated before flow cytometry analysis. To assess the effect of ER and mitochondrial contacts, HEK293T cells were transfected with VAPB/PTPIP51 or pcDNA as mock control. Twenty-four hours after transfection, cells were transfected with CLCC1-GFP(1-10)-HA and PIGBOS-3 × GFP11-FLAG. GFP signals were measured 48 h after transfection. Flow cytometry data were analyzed by FlowJo V10 software.

### Generation of PIGBOS-KO cells

PIGBOS-KO HEK293 and U2OS cells were generated according to the protocol described^53^. Briefly, cells were co-transfected with two CRISPR-Cas9 constructs with sgRNAs targeting at different PIGBOS gene loci. Twenty-four hours after transfection, GFP and mCherry double positive cells were isolated by FACS and single cells were sorted in a 96-well plate. PIGBOS-KO efficiency of single colonies was assessed by genotyping PCR, sequencing and Western blots. sgRNA and genotyping PCR primers are listed in Supplementary Table 5.

### Electron microscopy and analysis

Cells were cultured in 10-cm dishes and fixed with a solution of 2% paraformaldehyde and 2.5% glutaraldehyde in 0.1 M sodium cacodylate buffer with 2 mM calcium chloride at pH 7.4 for 15 minutes at room temperature (RT) followed by overnight fixation at 4 °C. Fixative was replaced with 0.1 M sodium cacodylate buffer (pH 7.4), and fixed cells were then carefully detached using an angled piece of Teflon held in a hemostat. Ribbons of cells were gently transferred into Eppendorf tubes. All samples were washed three times with 0.1 M sodium cacodylate buffer and then post-fixed in a solution of 2% osmium tetroxide reduced with 1.5% potassium ferrocyanide in 0.1 M sodium cacodylate buffer at room temperature for 40 min. All samples were gently pelleted between all steps. Samples were washed three times with distilled water and stained with 1% aqueous uranyl acetate for 40 min. The pellets were dehydrated in graded steps of ethanol before infiltration with Eponate 12 resin. Once the samples were fully infiltrated, they were centrifuged for 25 minutes at 8000 × *g* and polymerized at 60 °C for 48 h. Seventy-nanometers sections were cut with a Leica UC7 ultramicrotome using a diamond knife (Diatome). Images were acquired with a Zeiss Libra 120 EF-TEM with a 2 K CCD camera with 1 nm pixel size. To minimize bias in measurements, all EM images were acquired, segmented, and analyzed in a blinded fashion. Only cells with intact plasma membrane profiles were selected for imaging by the blinded technician.

After the images were acquired, the MOM and nearest ER membrane surfaces were manually segmented using the TrakEM2 plugin in the Fiji/ImageJ software environment^54^. For ER-MOM contact measurements, we used custom Python software to generate dilated boundaries of the MOM up to 30 nm away from the MOM in increments of 1 nm. The software then measured the total length of ER (*L*_*i*_) within each MOM dilation boundary *i* = 1, …, 30. The amount of ER surface within the region between two dilated boundaries was calculated as *l*_*i*_ = *L*_*i*_ − *L*_*i*−1_. The length L of the total ER-MOM contact was calculated as ${L} = \mathop {\sum}\nolimits_{{i} = 1}^{{i} = 30} {{l}_{i}}$. The average ER-MOM distance D was then calculated as the product of the contact length *l*_*i*_ and the corresponding dilation distance *i*, divided by the total length L (Eq. 1). The ER-MOM contact coefficient ERMICC^36^ was calculated as the contact length *L* divided by the product of the mitochondrial perimeter *P* and average distance *D* (Eq. 2). The p-value was calculated using the two tailed unpaired t-test.

1 $${D} = \frac{{\mathop {\sum }\nolimits_{{i} = 1}^{{i} = 30} {i} \times {l}_{i}}}{{\mathop {\sum }\nolimits_{{i} = 1}^{{i} = 30} {l}}_{\mathrm{i}}}$$

2 $${\mathrm{ERMICC}} = \frac{{L}}{{{P} \times {D}}}$$

### XBP-1 mRNA splicing assay

XBP-1 mRNA splicing assay was performed as previously described^55^. In brief, total RNA was extracted using PureLink RNA mini kit (Life Technologies, 12183025) and reverse transcribed to cDNA using QuantiTect Reverse Transcription kit (Qiagen, 205313). PCR primers 5′-CGGAAGCCAAGGGGAATGAAG-3′ and 5′-GGATATCAGACTCTGAATC-3′ encompassing the spliced sequences in XBP-1 mRNA were used for the RT-PCR amplification with Phusion HSII polymerase (Thermo, F565). RT-PCR products were separated by electrophoresis on a 2.5% agarose gel and visualized by ethidium bromide staining. GAPDH was used as the loading control with primers 5′-CATGTTCCAATATGATTCCACC-3′ and 5′-CTCCACGACGTACTCAGCG-3′.

### ATF6 luciferase assay

HEK293 cells were plated in a 6-well plate. The second day, cells were transfected with PIGBOS siRNA or non-targeting negative siRNA as indicated using Lipofectamine RNAi MAX. Meanwhile, cells were also transfected with PIGBOS-FLAG or pcDNA3.1(+) empty vector using Lipofectamine 2000. Twenty-four hours after transfection, cells were co-transfected with p5x-ATF6-GL3 and β-galactosidase. Six hours after transfection, cells were re-seeded in a 96 well plate, which was pre-treated with 50 µg/mL poly-L-lysine (Sigma, P1399) and incubated overnight. The next morning, cells were treated with indicated concentrations of TM for 24 h before measuring luciferase activities.

### Caspase-3 activity assay

PIGBOS-KD, PIGBOS-KO, and control U2OS cells were treated with indicated concentrations of TM or TG for 27 h. Caspase-3 activities were measured using EnzChekTM Caspase-3 Assay Kit (Life Technologies, E13183). Meanwhile, total cell lysate of each condition was analyzed by Western blotting using cleaved Caspase-3 and cleaved PARP antibodies.

### Cell viability (MTT) assay

U2OS cells were transfected with PIGBOS siRNA and negative control non-targeting siRNA using Lipofectamine RNAiMAX. Forty-eight hours after transfection, cells were treated with indicated concentration of TG for 48 h before viability measurement by the MTT assay method. Briefly, 3-(4,5-dimethylthiazol-2-yl)-2,5-diphenyltetrazolium bromide (MTT, EMD Millipore, 475989) was dissolved in PBS at a concentration of 5 mg/mL (10×) and then 1:10 diluted in DMEM + 10% FBS without phenol red (1x). Cell culture medium was replaced with 1 × MTT solution, and cells were incubated at 37 °C for 4 h. The medium was then aspirated, and DMSO was added to dissolve the insoluble formazan product. Absorbance at 570 nm was measured using a BioTek Synergy H5 microplate reader.

### Structure analysis for PIGBOS microprotein

PIGBOS structure was analyzed by inputting the PIGBOS microprotein sequence into the TMDOCK server^43^ (https://membranome.org/tmdock). The predicted structure was illustrated by PyMol.

## Supplementary information

Supplementary Information

Description of Additional Supplementary Files

Supplementary Data 1

Source Data

## Data Availability

The data supporting the findings are available within the article and Supplementary Information. RNA-Seq and Ribosome profiling data for PIGBOS (Supplementary Fig. 1a) are analyzed from a study that will be published separately, and the data have been deposited into Gene Expression Ominbus database with accession number GSE125218 (https://www.ncbi.nlm.nih.gov/geo/query/acc.cgi?acc=GSE125218). The MS data for PIGBOS-FLAG immunoprecipitation have been deposited to the ProteomeXchange Consortium via the PRIDE partner repository with the dataset identifier PXD014890. All other data are available from the authors upon reasonable request. The source data underlying Figs. 4a, d, e, 5b, 6c–e, 6g and Supplementary Figs. 13b, d, 14a, b, 15c, d, 16a, b, 17a, c, d, 18a, b and 20a–c are provided as a Source Data file.
