# Supplementary material for: Regulation of the ER stress response by a mitochondrial microprotein
Source: Nat Commun. 2019 Oct 25;10:4883. doi: 10.1038/s41467-019-12816-z (PMC6814811; doi:10.1038/s41467-019-12816-z)
Supplement: Supplementary file 1 — Supplementary Information [file 41467_2019_12816_MOESM1_ESM.pdf]

Supplementary Information

Regulation of the ER stress response by a mitochondrial microprotein

Qian Chu[1], Thomas F. Martinez[1], Sammy Weiser Novak[2], Cynthia J. Donaldson[1], Dan Tan[1], Joan M. Vaughan[1], Tina Chang[1], Jolene K. Diedrich[1], Leo Andrade[2], Andrew Kim[1], Tong Zhang[2], Uri Manor[2,*], Alan Saghatelian[1,*]

Correspondence to: umanor@salk.edu and asaghatelian@salk.edu

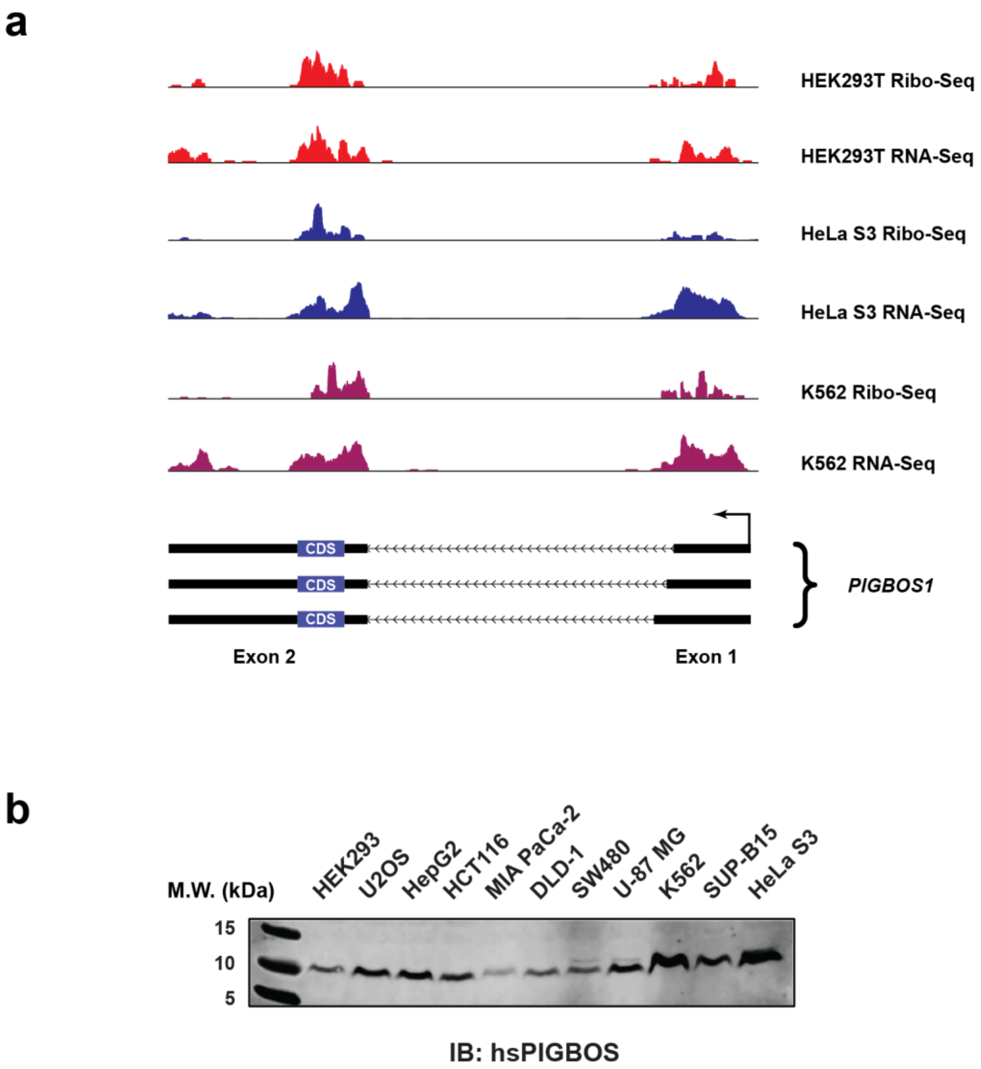

Supplementary Fig. 1. PIGBOS is endogenously expressed in several human cell lines. a, The PIGBOS transcript consists of two exons and has three splice isoforms in human with slight differences in the first exon and the same second exon that contains the PIGBOS microprotein encoding smORF. RNA-Seq and Ribo-Seq analysis of HEK293T, HeLa S3, and K562 cells reveal that PIGBOS is robustly expressed. **b**, 50 µg of total cell lysate from indicated human cell lines were separated by 4-12% SDS-PAGE gel and analyzed by anti-human PIGBOS antibody.

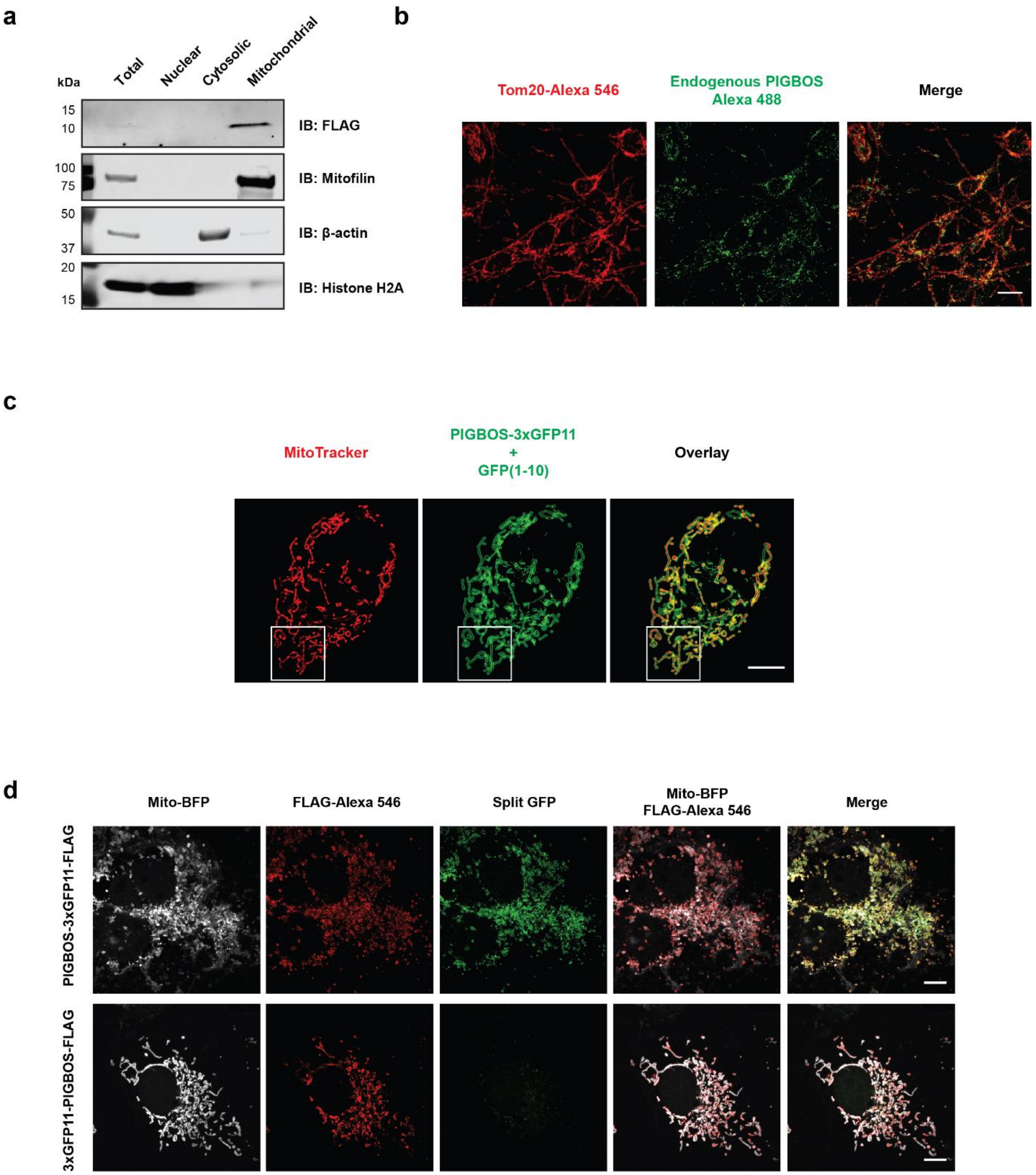

Supplementary Fig. 2. PIGBOS is a mitochondrial outer membrane microprotein with its N-terminal transmembrane domain embedded in the MOM and C-terminus facing the cytoplasm. a, HEK293T cells were transfected with PIGBOS-FLAG and nuclear, cytosolic and mitochondrial fractions were isolated. 20 μg of each fraction was analyzed by Western blot with indicated subcellular markers and FLAG antibody. **b**, Confocal imaging using anti-PIGBOS antibody reveals co-localization of endogenous PIGBOS with mitochondria in rat C6 cells. Scale

bar: 10 µm. **c**, COS-7 cells were co-transfected with PIGBOS-3×GFP11 and GFP (1-10). 48 hours after transfection, mitochondria were labeled using MitoTracker Deep Red FM. Cells were then imaged at 37ºC and 5% CO2 using a Zeiss LSM 880 Airyscan confocal microscope. The region in the white box was zoomed in and re-imaged at higher resolution as shown in Fig. 2d. Scale bar: 10 µm. **d**, COS-7 cells were co-transfected with GFP (1-10) and PIGBOS-3×GFP11-FLAG or 3×GFP11-PIGBOS-FLAG. 24 hours later, cells were transfected with mito-BFP to label mitochondria. 24 hours after transfection, cells were fixed and stained with anti-FLAG antibody overnight before imaging. Scale bar: 10 µm.

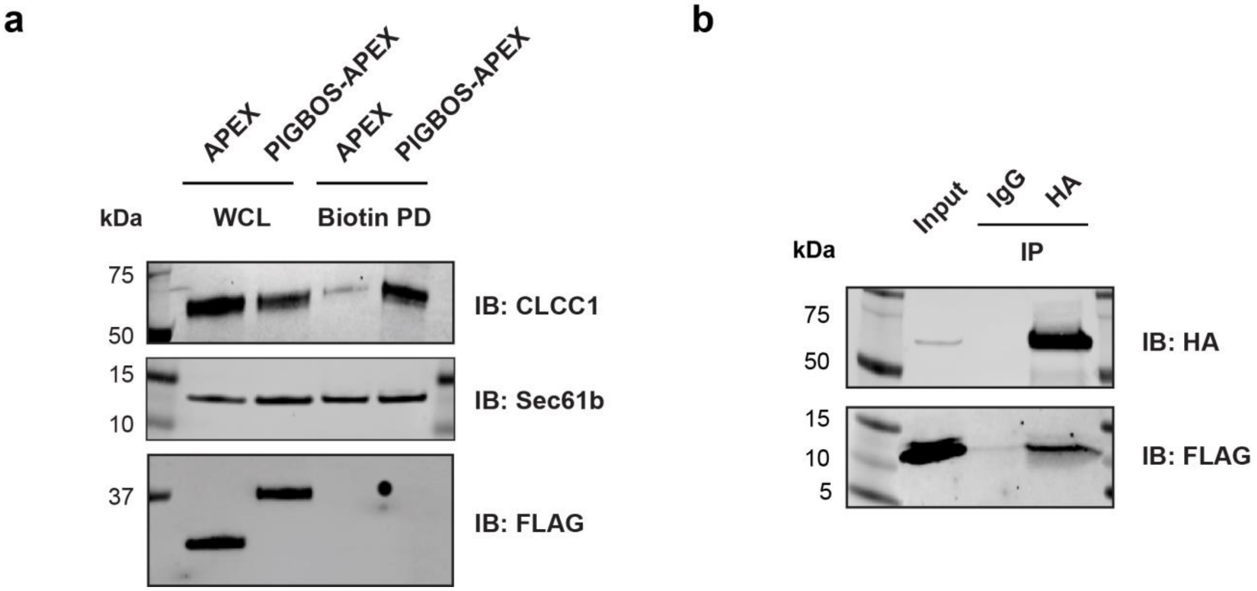

Supplementary Fig. 3. Validation of PIGBOS-CLCC1 interaction. a, Proximity labeling by PIGBOS-APEX indicated that CLCC1 is specifically biotinylated and enriched, whereas the ER marker Sec61b is equally biotinylated by PIGBOS-APEX and APEX control. **b**, Reciprocal co-immunoprecipitation of CLCC1-HA and PIGBOS-FLAG in HEK293T cells revealed that PIGBOS-FLAG co-elutes with CLCC1-HA.

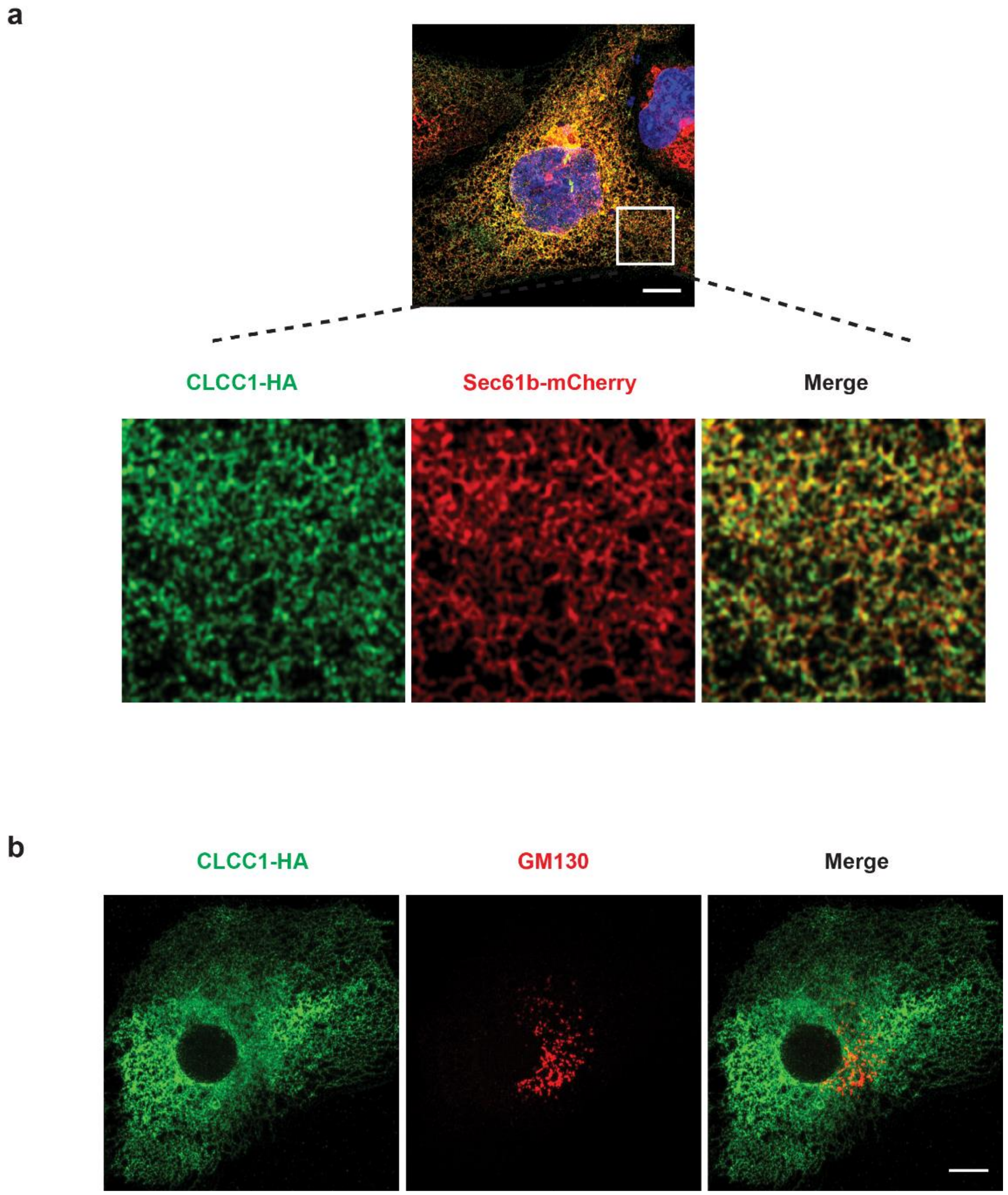

Supplementary Fig. 4. Localization of CLCC1 protein. a, Confocal imaging of CLCC1-HA in COS-7 cells showed that it overlaps with the ER marker Sec61b-mCherry. Scale bar: 10 µm. **b**, Confocal imaging of CLCC1-HA in U2OS cells showed that it does not colocalize with the Golgi marker GM130. Scale bar: 10 µm.

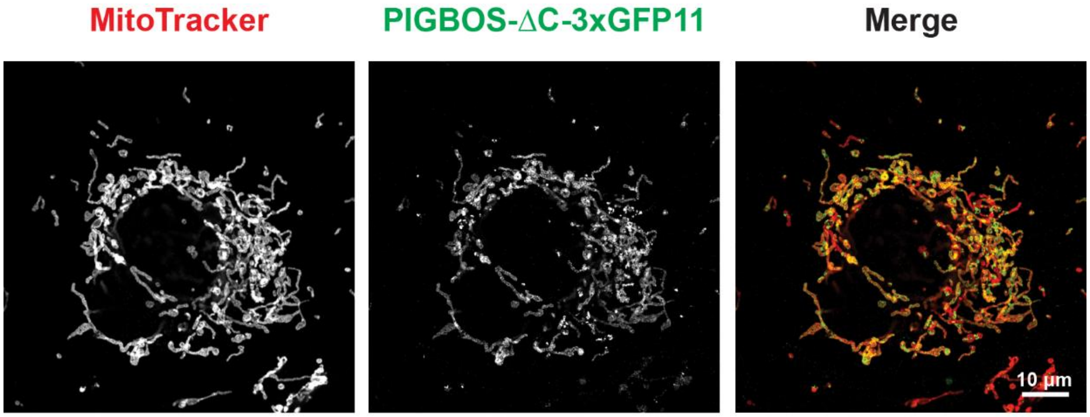

Supplementary Fig. 5. Mitochondrial localization of PIGBOS-ΔC-3×GFP11. The subcellular localization of PIGBOS-ΔC-3×GFP11 was assessed by transfection of PIGBOS-ΔC-3×GFP11-FLAG into COS-7 cells and detection with anti-FLAG immunofluorescence. MitoTracker Red CMXRos was used to label the mitochondria.

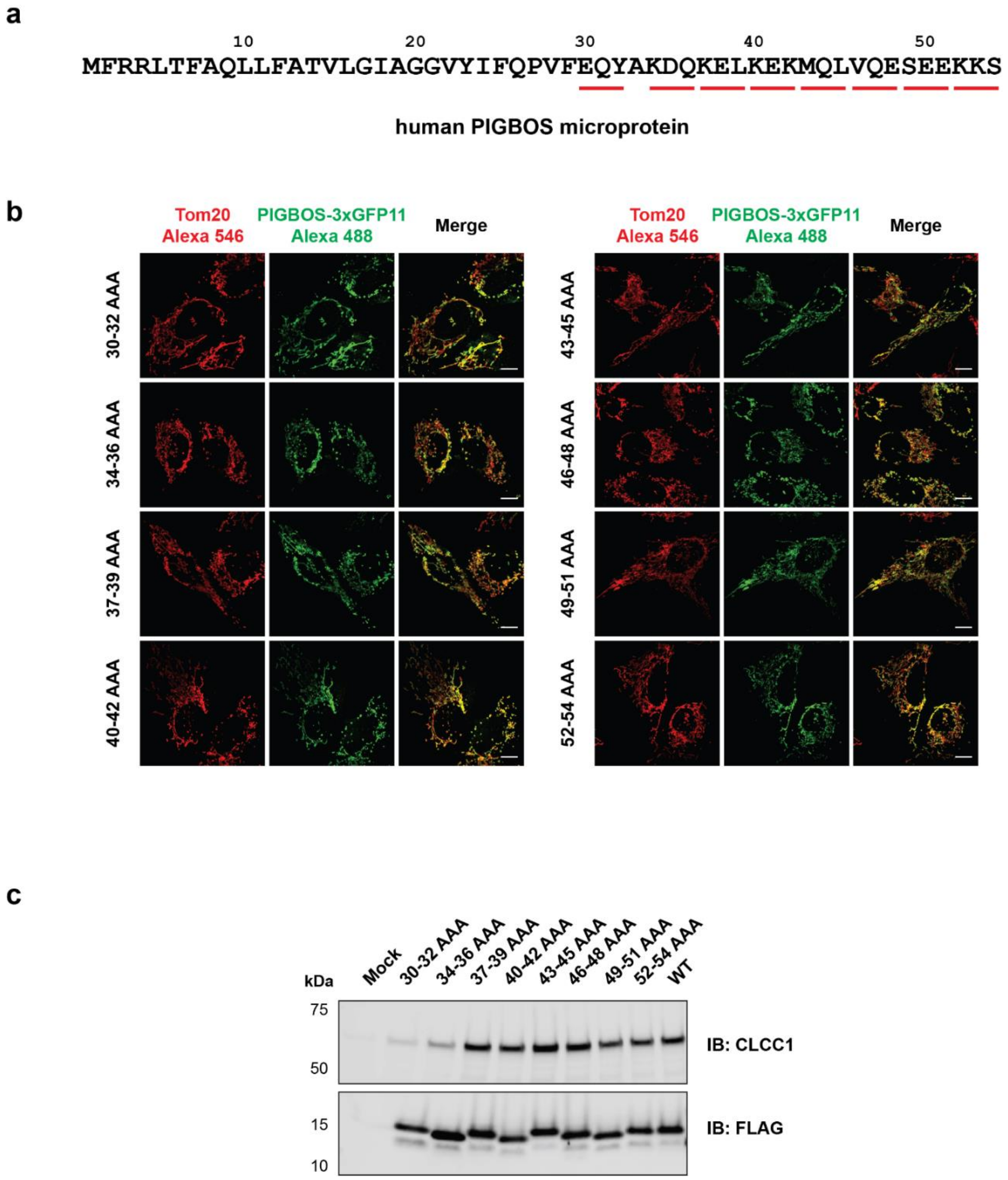

Supplementary Fig. 6. The C-terminal region (C-Term) of PIGBOS is required for the CLCC1 interaction. a, Triple alanine mutant scanning of PIGBOS cytosolic domain (aa 30-54) in full-length PIGBOS-3×GFP11-FLAG was performed to identify residues essential for the interaction with CLCC1. Every three consecutive residues that are mutated to alanine are underlined in red. **b**, Confocal imaging of triple alanine mutants in U2OS cells indicated that they are localized in mitochondria as indicated by overlapping with the mitochondrial marker Tom20. Scale bars: 10 µm. **c**, The mutant constructs were expressed in HEK293T cells, and subjected to anti-FLAG immunoprecipitation followed by anti-CLCC1 Western blotting to evaluate PIGBOS-CLCC1 interaction.

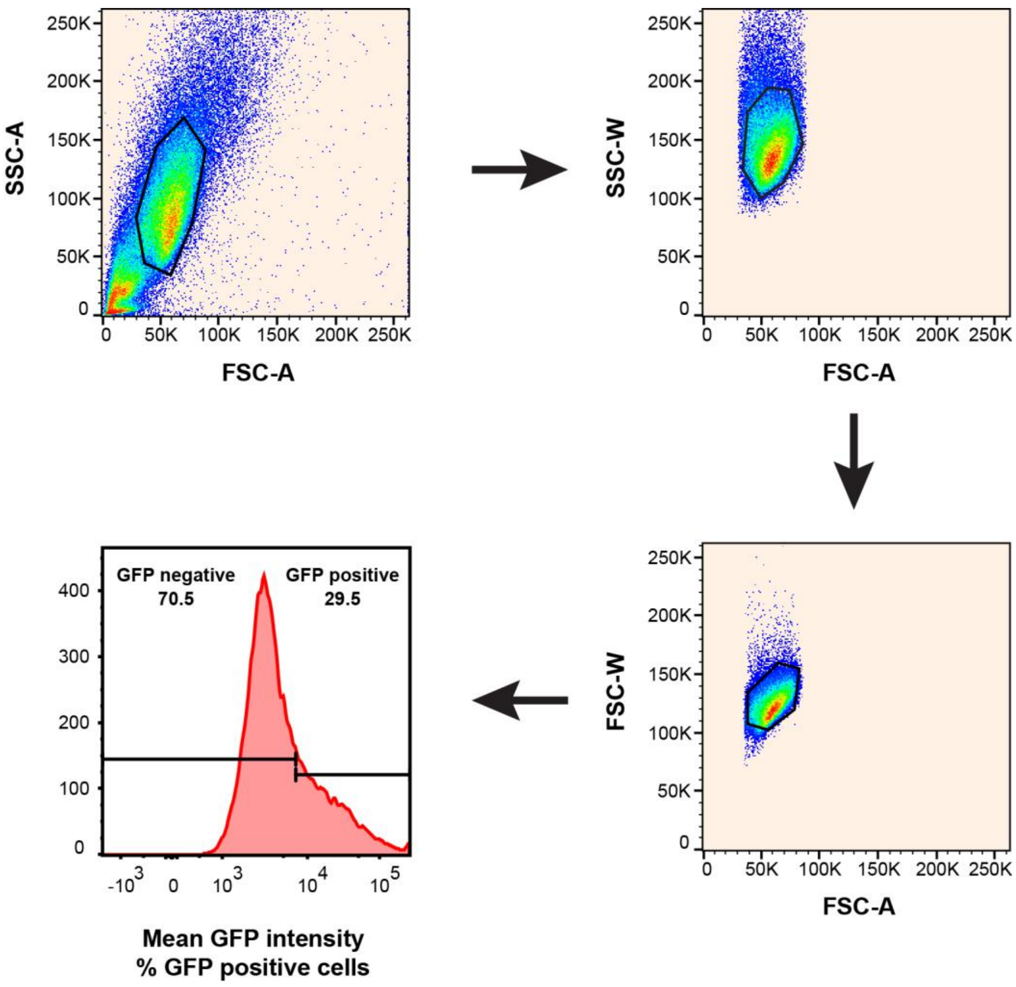

Supplementary Fig. 7. Gating strategy used for flow cytometry experiments in this study.

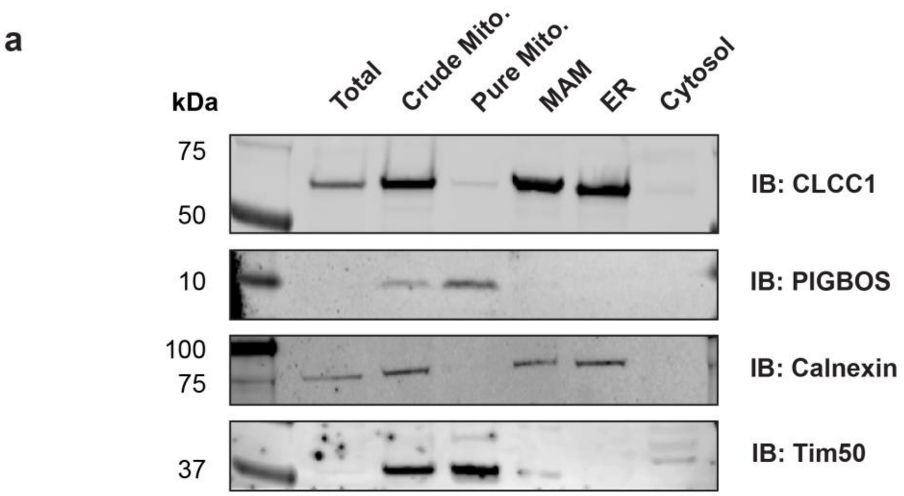

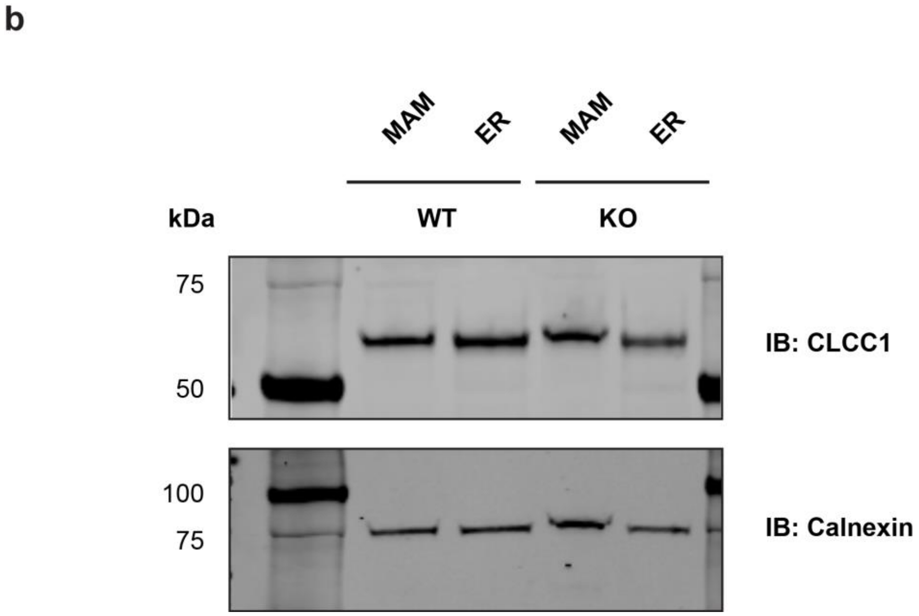

Supplementary Fig. 8. Localization of CLCC1 in mitochondria associated membranes (MAM). a, Subcellular fractionation of HEK293T cells revealed that CLCC1 is present in the ER and MAM, and PIGBOS is located in the mitochondria, with Calnexin and Tim50 serving as ER/MAM and mitochondrial markers, respectively. **b**, Western blots of MAM and ER fractions from HEK293 WT and PIGBOS-KO cells revealed similar CLCC1 localizations.

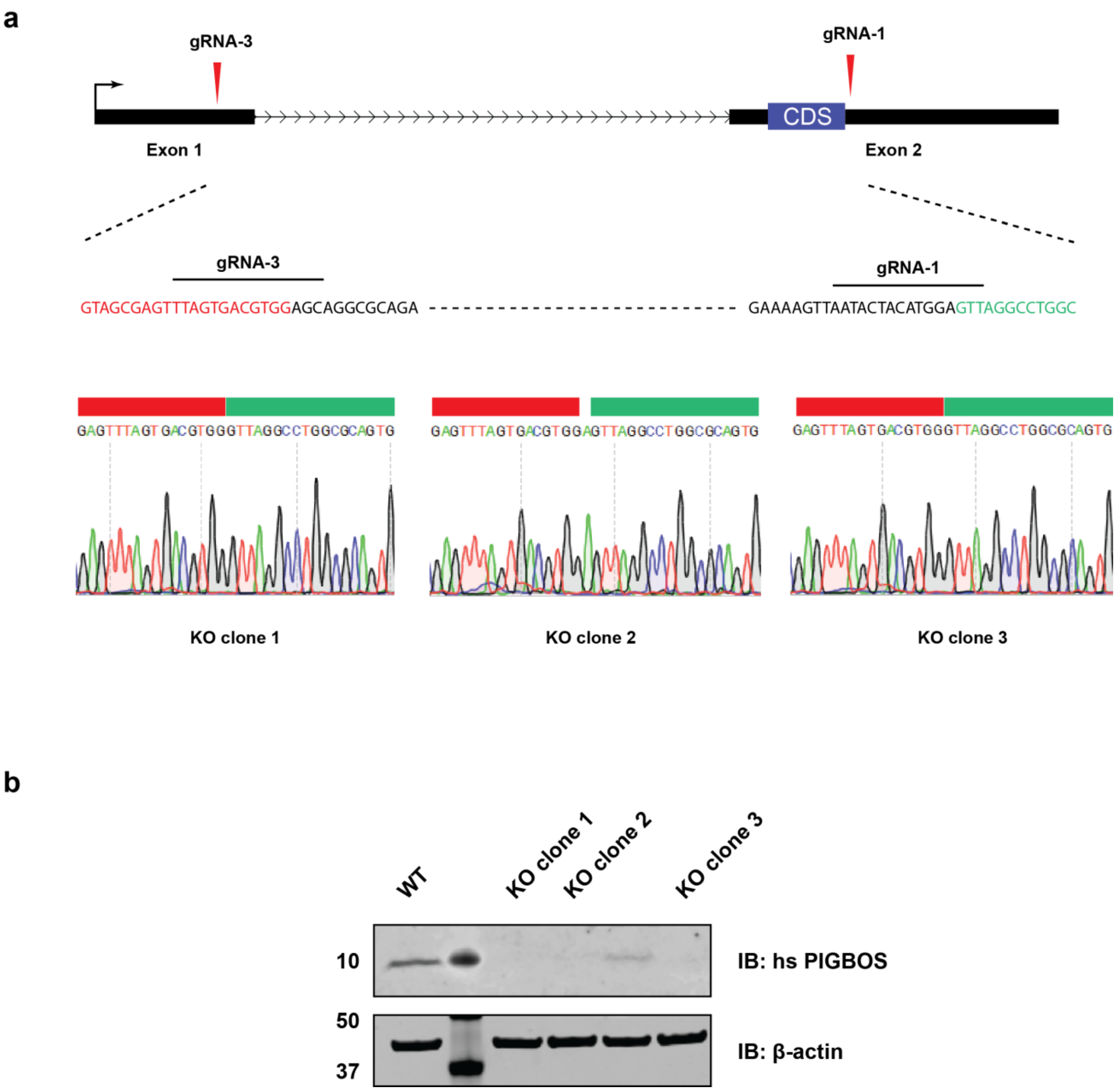

Supplementary Fig. 9. Generation of PIGBOS-KO U2OS cells. a, Top, an illustration of gRNA sequences and targeting sites in *PIGBOS1* gene. Bottom, sequencing results of three PIGBOS-KO single clones. **b**, Western blots of WT and PIGBOS-KO cells confirm the ablation of PIGBOS in U2OS cells.

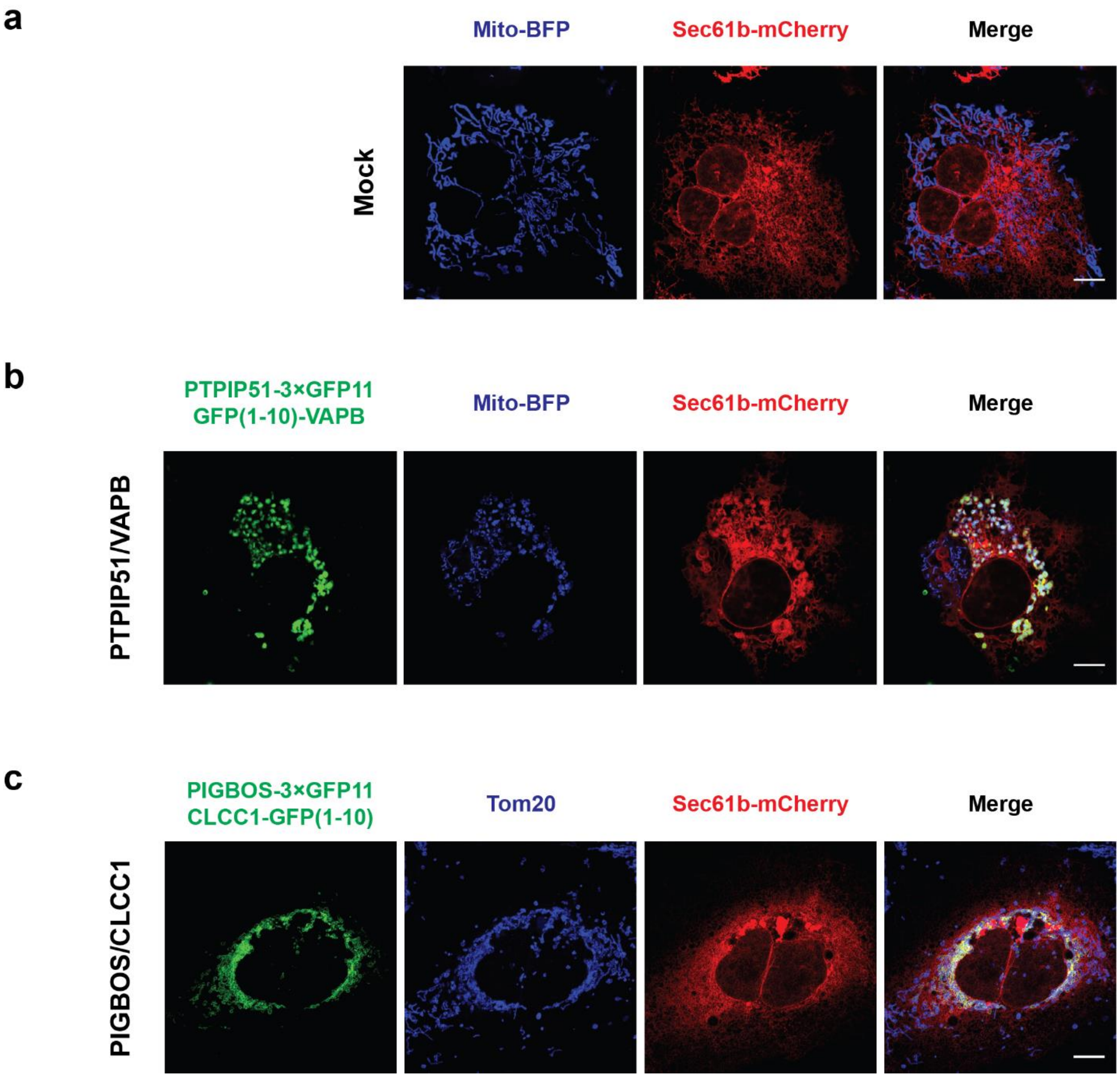

Supplementary Fig. 10. PIGBOS-CLCC1 bimolecular complementation does not affect ER-mitochondria morphology. a, COS-7 cells were transfected with mito-BFP and Sec61b-mCherry, and subjected to live cell imaging 24 hours after transfection. **b**, COS-7 cells were co-transfected with PTPIP51-3×GFP11-FLAG and HA-GFP(1-10)-VAPB. 24 hours later, cells were transfected with mito-BFP and Sec61b-mCherry to label mitochondria and ER respectively. Cells were analyzed by live cell imaging post-transfection. **c**, COS-7 cells were co-transfected with PIGBOS-3×GFP11 and CLCC1-GFP(1-10). 24 hours later, cells were transfected with Sec61b-mCherry to label ER. Cells were then fixed and stained with anti-Tom20 antibody overnight before imaging. Scale bars: 10 μm.

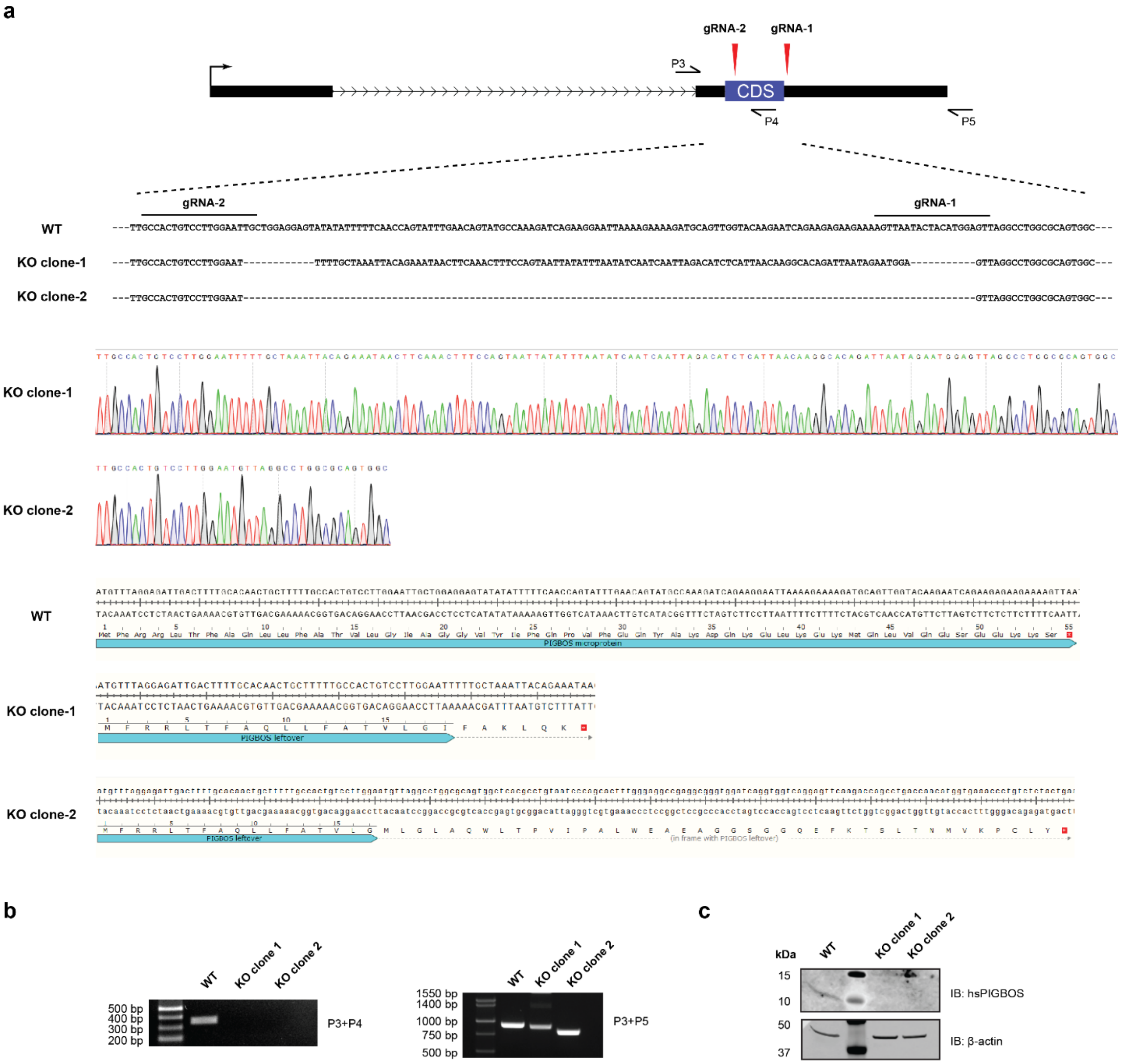

Supplementary Fig. 11. Generation of PIGBOS-KO HEK293 cells. a, Top, an illustration of gRNA targeting sites in *PIGBOS1* gene. Middle, DNA sequencing results of PIGBOS-KO clones. Bottom, Comparison of PIGBOS WT protein sequence with KO clones. **b**, PCR of gDNA from WT and PIGBOS-KO cells with primers indicated in (**a**). **c**, Western blots of PIGBOS WT and KO cells confirm the ablation of PIGBOS in HEK293 cells.

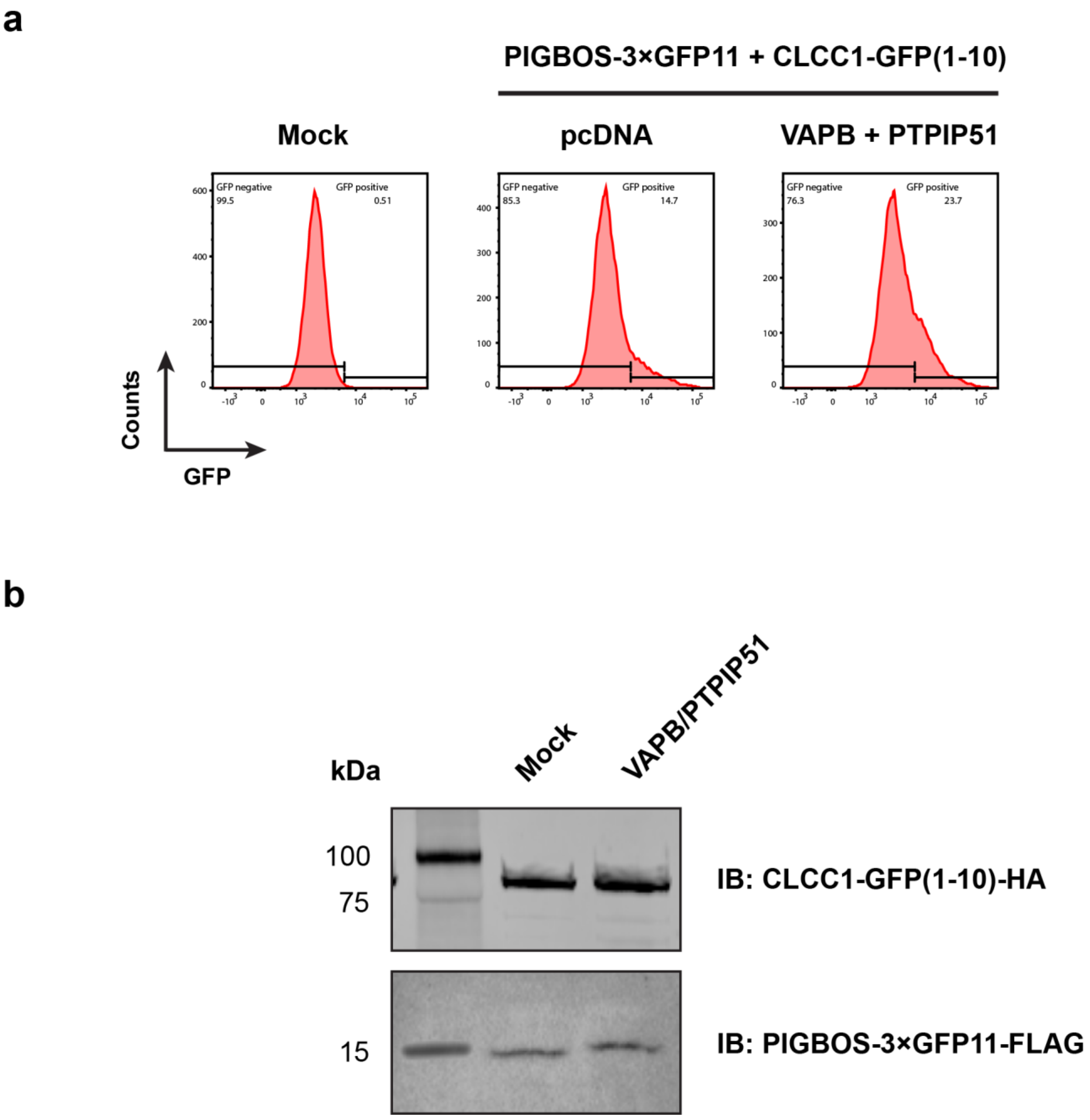

Supplementary Fig. 12. Regulation of PIGBOS-CLCC1 interaction with increased ER-mitochondria contacts. a, Flow cytometry measurement of PIGBOS-CLCC1 interaction in HEK293T cells expressing VAPB/PTPIP51. HEK293T cells were co-transfected with VAPB and PTPIP51, and PIGBOS-3×GFP11-FLAG and CLCC1-GFP(1-10)-HA were transfected 24 hours later. GFP signals were assessed by flow cytometry 48 hours after transfection. **b**, Western blots of PIGBOS-3×GFP11-FLAG and CLCC1-GFP(1-10)-HA in whole cell lysate indicated that the two proteins express at a similar level in control and VAPB/PTPIP51 expressing cells.

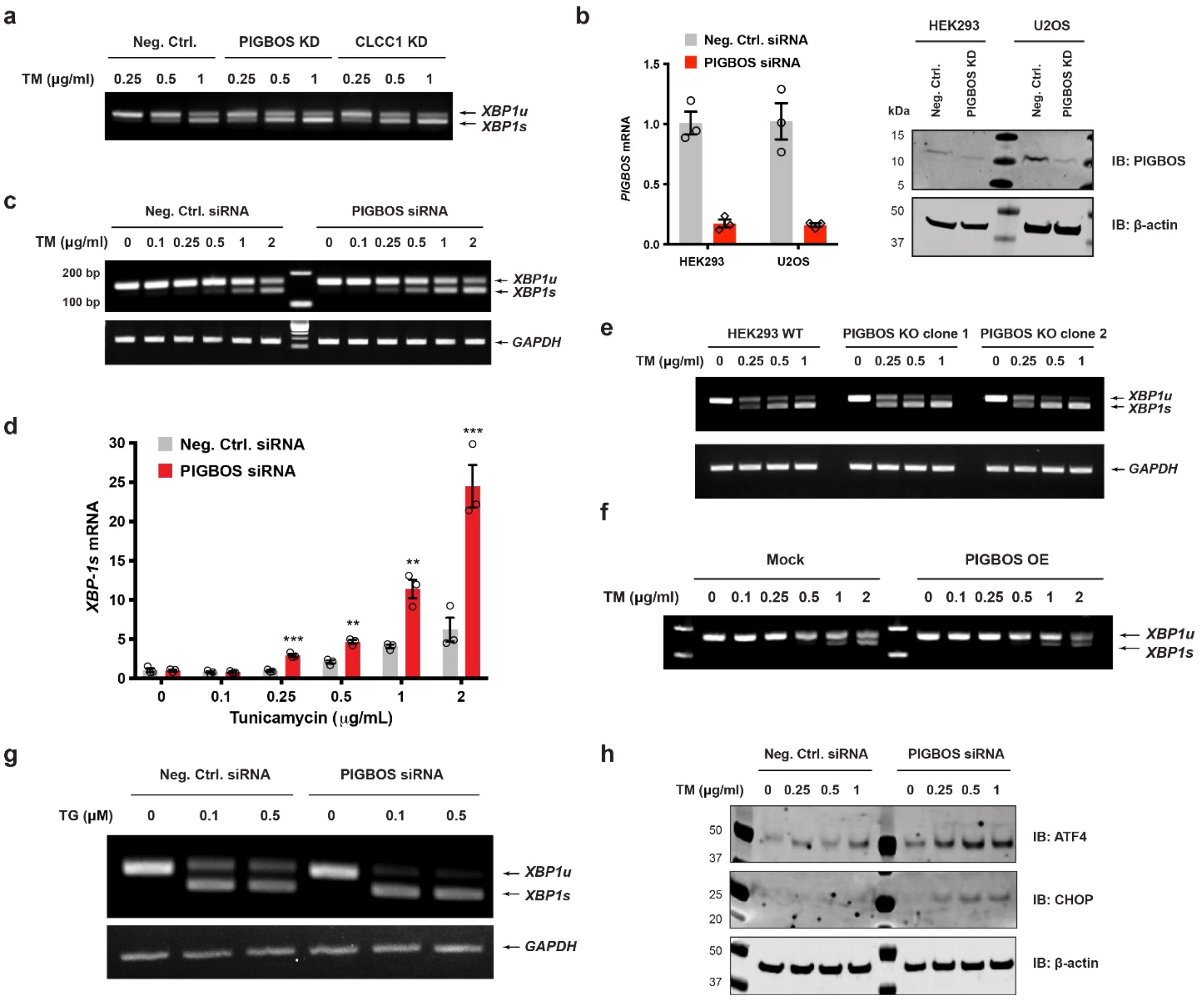

Supplementary Fig. 13. PIGBOS regulates unfolded protein responses. a, PIGBOS-KD, CLCC1-KD and control HEK293 cells were treated with indicated concentrations of TM for 3 hours. XBP-1 mRNA splicing was determined by RT-PCR of total cDNA from cells for each condition. **b**, HEK293 and U2OS cells were transfected with siRNA targeting PIGBOS or negative control siRNA for 48 hours. PIGBOS knockdown efficiency was demonstrated by RT-qPCR (left) and Western blots (right). **c** and **d**, PIGBOS-KD and control HEK293 cells were treated with indicated doses of TM for 3 hours. XBP-1 mRNA splicing was assessed by RT-PCR (**c**) and RT-qPCR quantification of spliced XBP-1 (XBP1s) (**d**). **e**, XBP-1 mRNA splicing was measured in PIGBOS-KO and WT HEK293 cells by RT-PCR. **f**, RT-PCR analysis of XBP-1 mRNA splicing in PIGBOS overexpressed and mock-transfected HEK293 cells treated with indicated doses of TM for 3 hours. **g**, XBP-1 mRNA splicing was measured in PIGBOS-KD and control HEK293 cells treated with indicated doses of TG for 3 hours. **h**, Western blots of ATF4 and CHOP in PIGBOS-KD and control HEK293 cells treated with indicated concentrations of TM for 24 hours. β-actin was used as a loading control. Error bars, s.e.m.. The *p*-values were calculated using Student's t-test, $**p < 0.01$, $***p < 0.001$, n = 3. Source Data for Supplementary Figs. 13b and 13d are provided as a Source Data file.

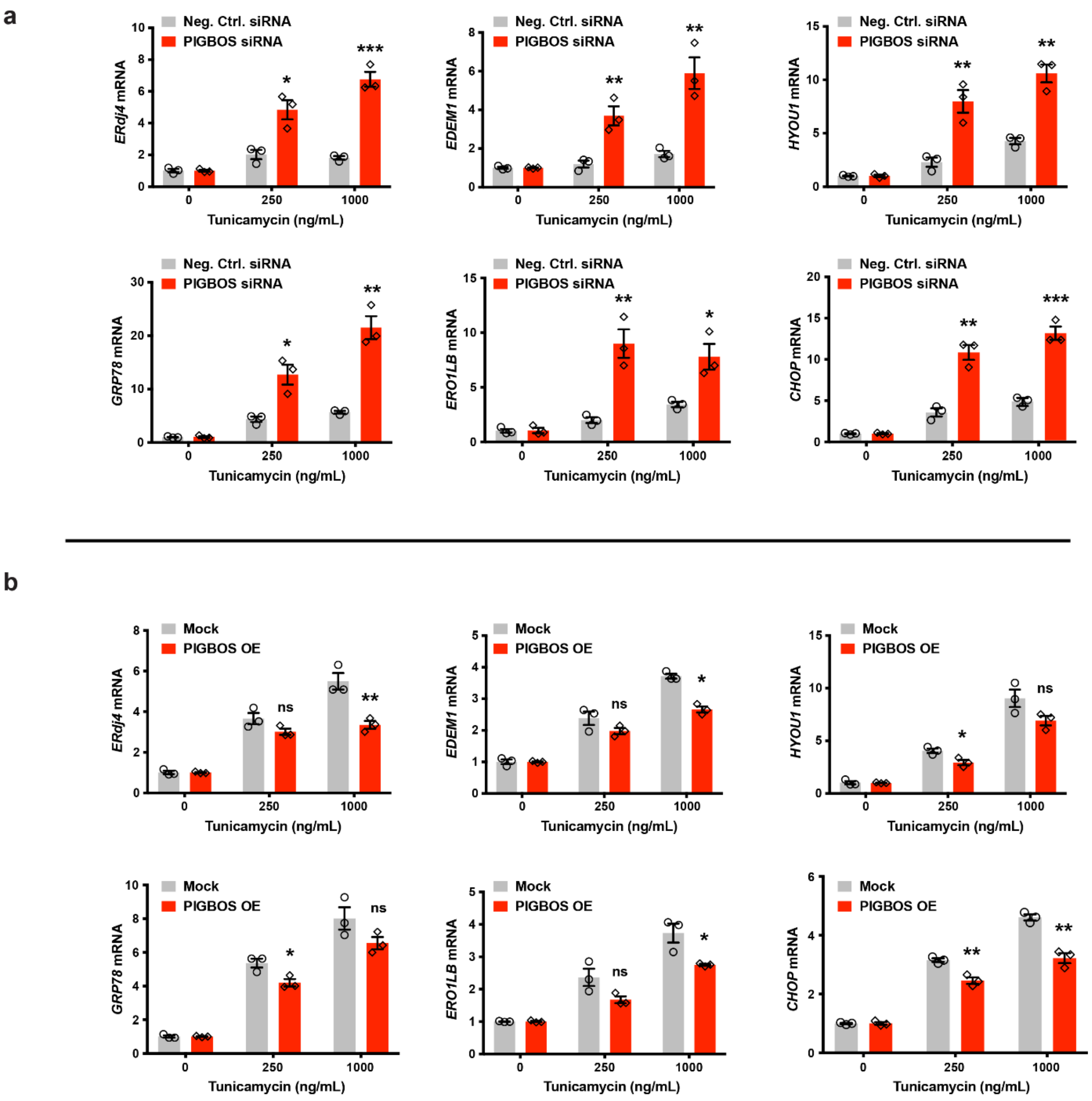

Supplementary Fig. 14. RT-qPCR analysis of UPR targeting genes in PIGBOS-KD and overexpressed HEK293 cells. PIGBOS-KD (**a**) and PIGBOS overexpressed (**b**) cells were treated with indicated concentrations of tunicamycin for 8 hours, and mRNA levels of UPR targeting genes (*ERdj4*, *EDEM1*, *HYOU1*, *GRP78*, *ERO1LB*, *CHOP*) were determined by RT-qPCR. Error bars, s.e.m.. The p-values were calculated using Student's t-test, ns = not significant, *$p < 0.05$, **$p < 0.01$, ***$p < 0.001$, n = 3. Source Data are provided as a Source Data file.

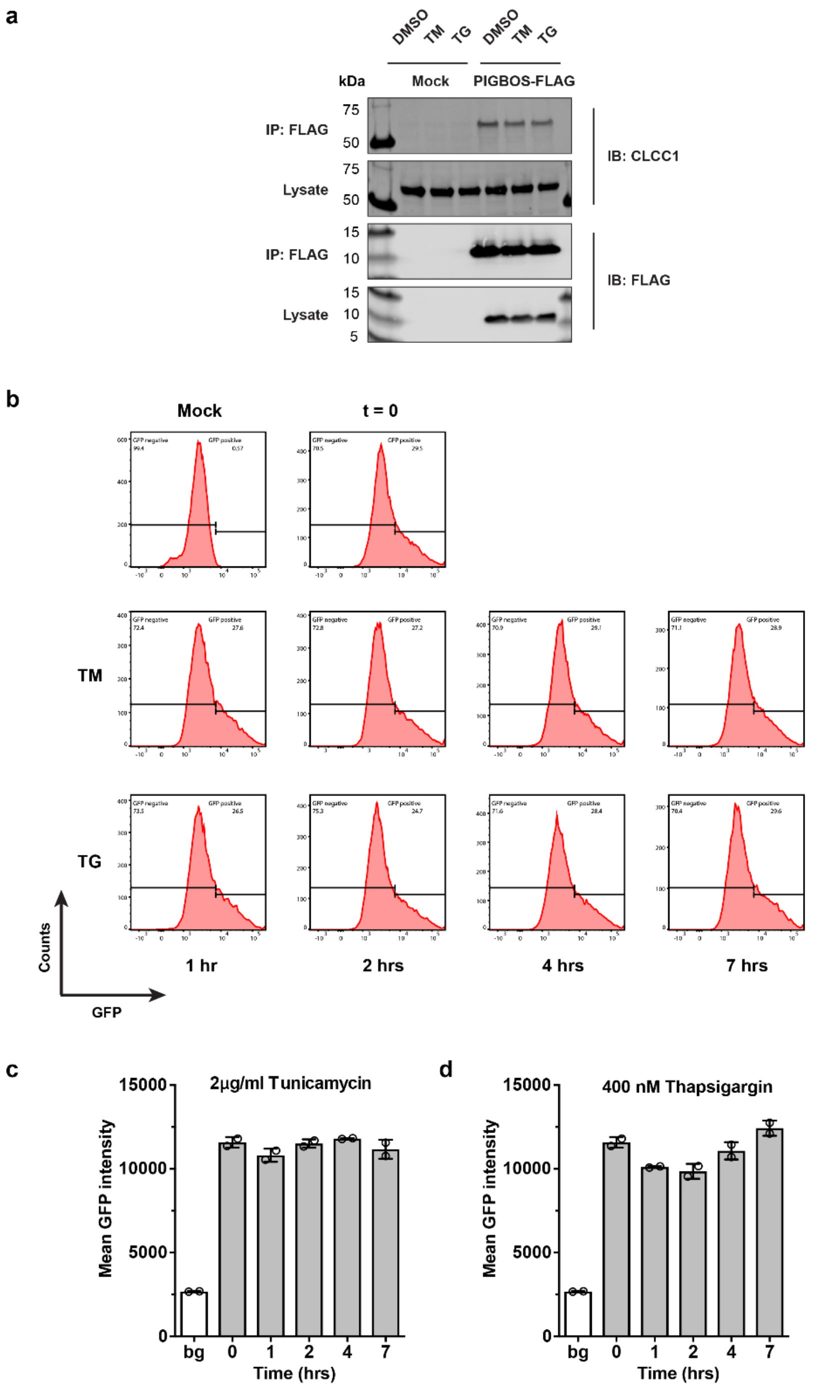

Supplementary Fig. 15. PIGBOS-CLCC1 interaction during ER stress. a, FLAG immunoprecipitation of PIGBOS-FLAG expressing HEK293T cells with treatment of tunicamycin (TM, 2 µg/ml) or thapsigargin (TG, 500 nM) for 4 hours. **b**, Flow cytometry analysis of HEK293T cells co-expressing PIGBOS-3×GFP11-FLAG and CLCC1-GFP(1-10)-HA after TM (2 µg/ml) or TG (400 nM) treatment as indicated. **c** and **d**, Quantification of PIGBOS-CLCC1 split GFP intensities in (**c**) TM and (**d**) TG treated cells over time. Error bars, s.d., n = 2. Source Data for Supplementary Figs. 15c and 15d are provided as a Source Data file.

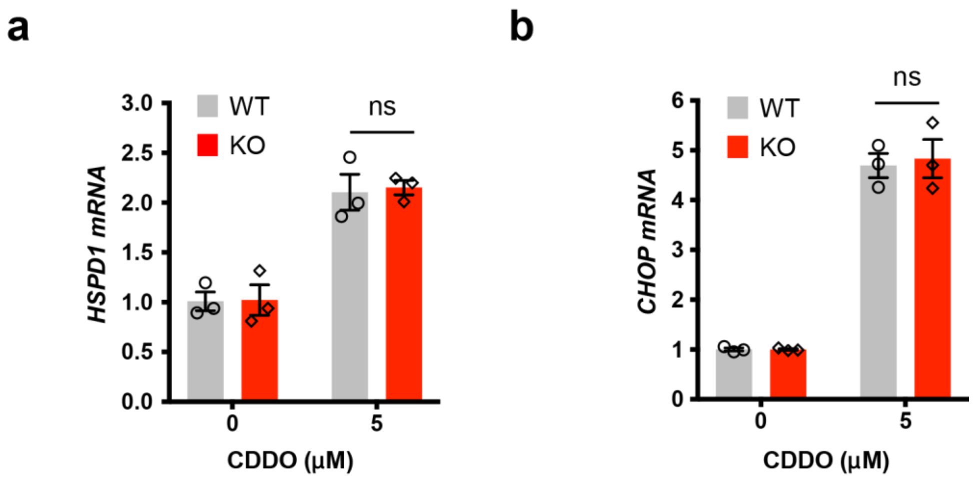

Supplementary Fig. 16. PIGBOS has no effect on mitochondrial UPR. RT-qPCR quantification of mitochondrial UPR targeting genes (**a**) *HSPD1* and (**b**) *CHOP* in PIGBOS-KO and WT HEK293 cells after 6-hour treatment with vehicle or 5 μM of bardoxolone (CDDO). Error bar, s.e.m.. The *p*-values were calculated using Student's t-test, ns = not significant, n = 3. Source data are provided as a Source Data file.

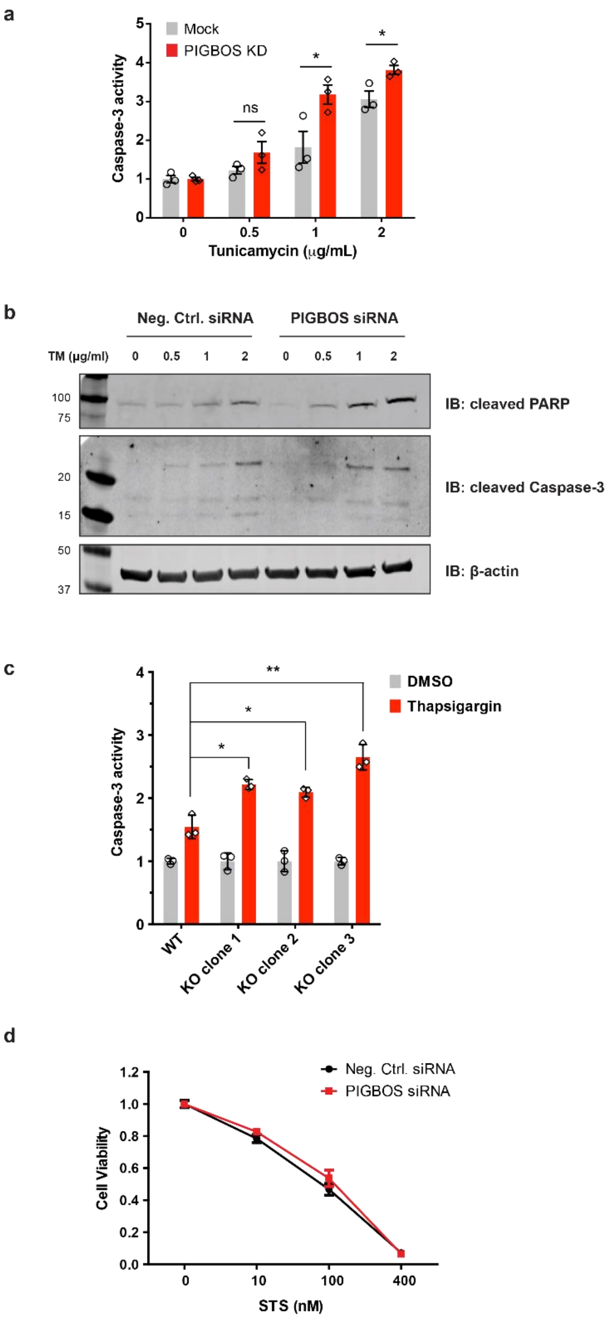

Supplementary Fig. 17. Loss of PIGBOS enhances caspase activity during prolonged ER stress. a and **b**, PIGBOS-KD and control U2OS cells were treated with indicated concentrations of Tunicamycin for 27 hours. Caspase-3 activities were determined by (**a**) a colorimetric assay and (**b**) Western blots of cleaved PARP and caspase-3. **c**, Caspase-3 activities were assessed in PIGBOS-KO and WT U2OS cells treated with 0.5 μM of Thapsigargin for 27 hours. **d**, PIGBOS-KD and control U2OS cells treated with indicated doses of staurosporine (STS) for 48 hours followed by cell viability measurements using MTT. Error bars, s.e.m.. The *p*-values were calculated using Student's t-test, ns = not significant, *$p < 0.05$, **$p < 0.01$, n = 3. Source Data for Supplementary Figs. 17a, 17c and 17d are provided as a Source Data file.

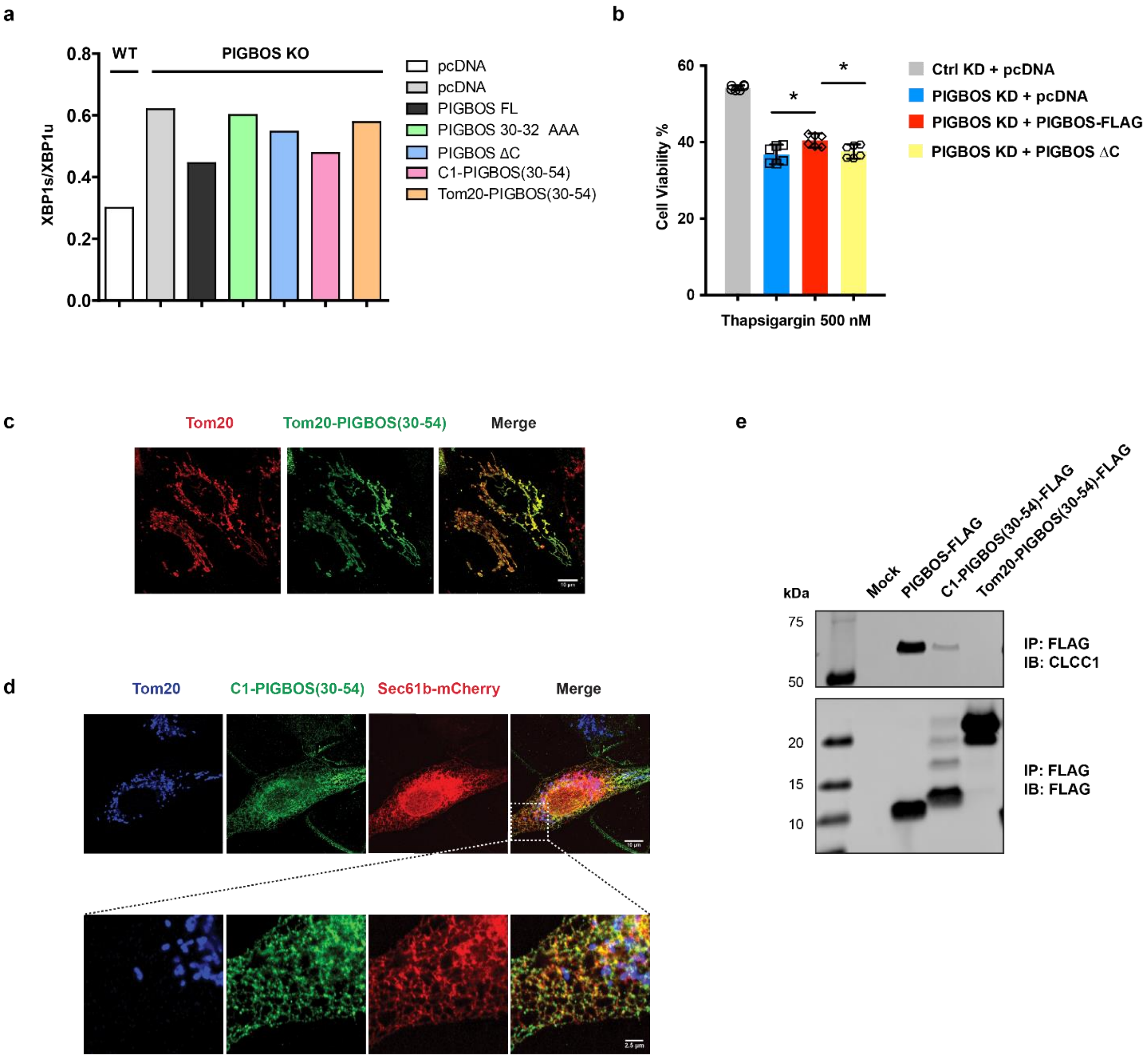

Supplementary Fig. 18. Binding and UPR activity of chimeric PIGBOS proteins. a, Illustration of XBP1 splicing activity by quantification of band intensities of unspliced and spliced XBP1 in Fig. 6h. **b**, U2OS cells were transfected with PIGBOS siRNA and negative control siRNA, and 24 hours later, cells were transfected with PIGBOS constructs as indicated. 24 hours after transfection, cells were treated with 500 nM of TG for 48 hours followed by cell viability measurements using MTT. (Error bars, s.d., the *p*-values were calculated using Student's t-test, *$p < 0.05$, n = 6.) **c** and **d**, Confocal imaging of Tom20-PIGBOS(30-54) and C1-PIGBOS(30-54) in U2OS cells. Cells were transfected with (**c**) Tom20-PIGBOS(30-54)-FLAG or (**d**) C1-PIGBOS(30-54)-FLAG and Sec61b-mCherry. 48 hours later, cells were fixed and stained with Tom20 and FLAG antibodies overnight before imaging. **e**, HEK293T cells were transfected with PIGBOS WT, C1-PIGBOS(30-54) and Tom20-PIGBOS(30-54). FLAG immunoprecipitation was performed 48 hours after transfection and indicated that these two chimeric proteins are not able to bind CLCC1. Source Data for Supplementary Figs. 18a and 18b are provided as a Source Data file.

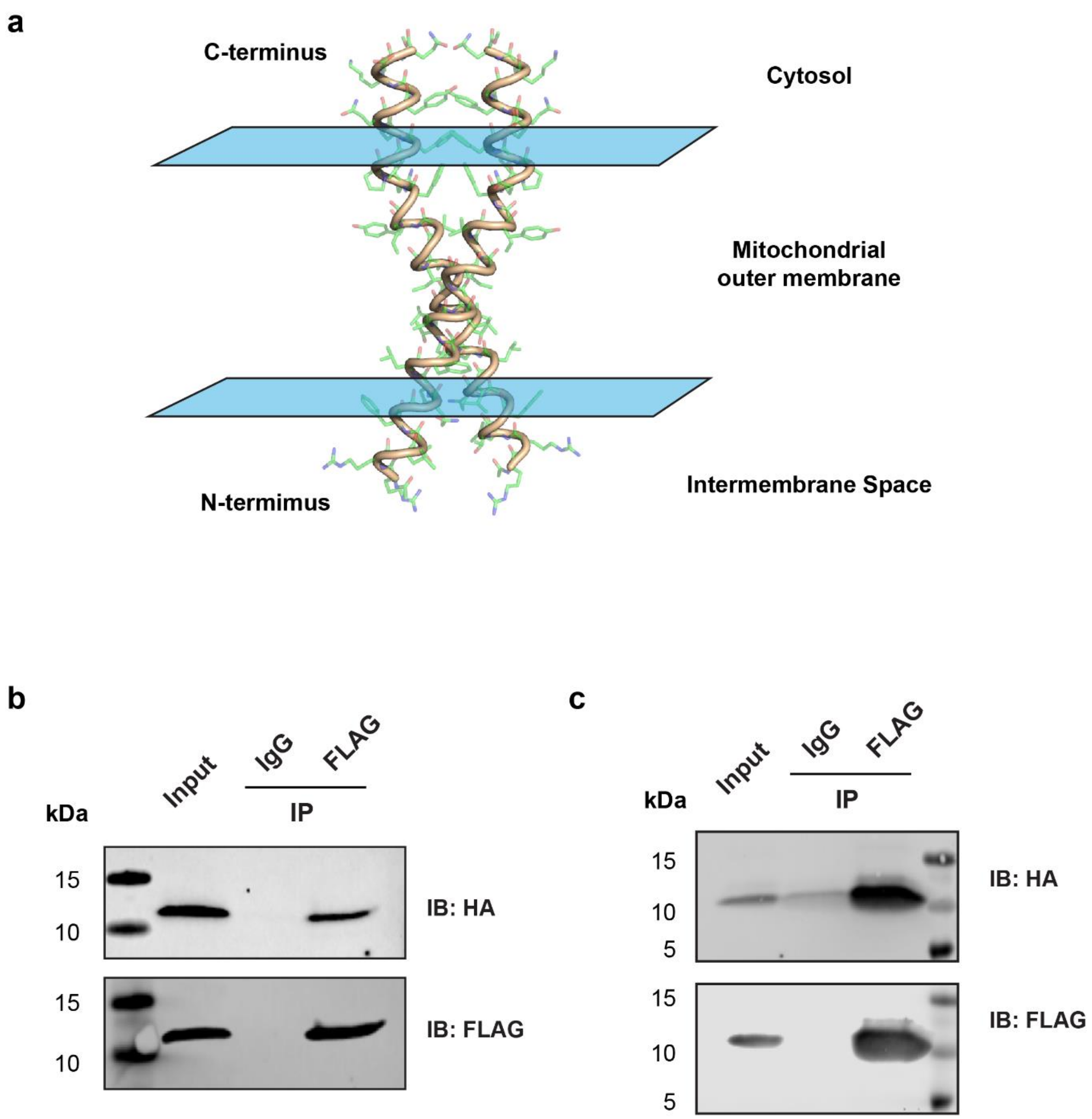

Supplementary Fig. 19. PIGBOS forms a dimeric structure. a, Analysis of PIGBOS sequence using the TMDOCK server predicts that PIGBOS TM region dimerizes. **b**, FLAG immunoprecipitation of HEK293T cell lysates containing human PIGBOS-FLAG and human PIGBOS-HA demonstrated an interaction between PIGBOS-FLAG and PIGBOS-HA indicating that PIGBOS is an oligomer. **c**, HEK293T cells were co-transfected with human PIGBOS-FLAG and rat PIGBOS-HA. Forty-eight hours after transfection, FLAG IP demonstrated that human PIGBOS associated the conserved rat PIGBOS.

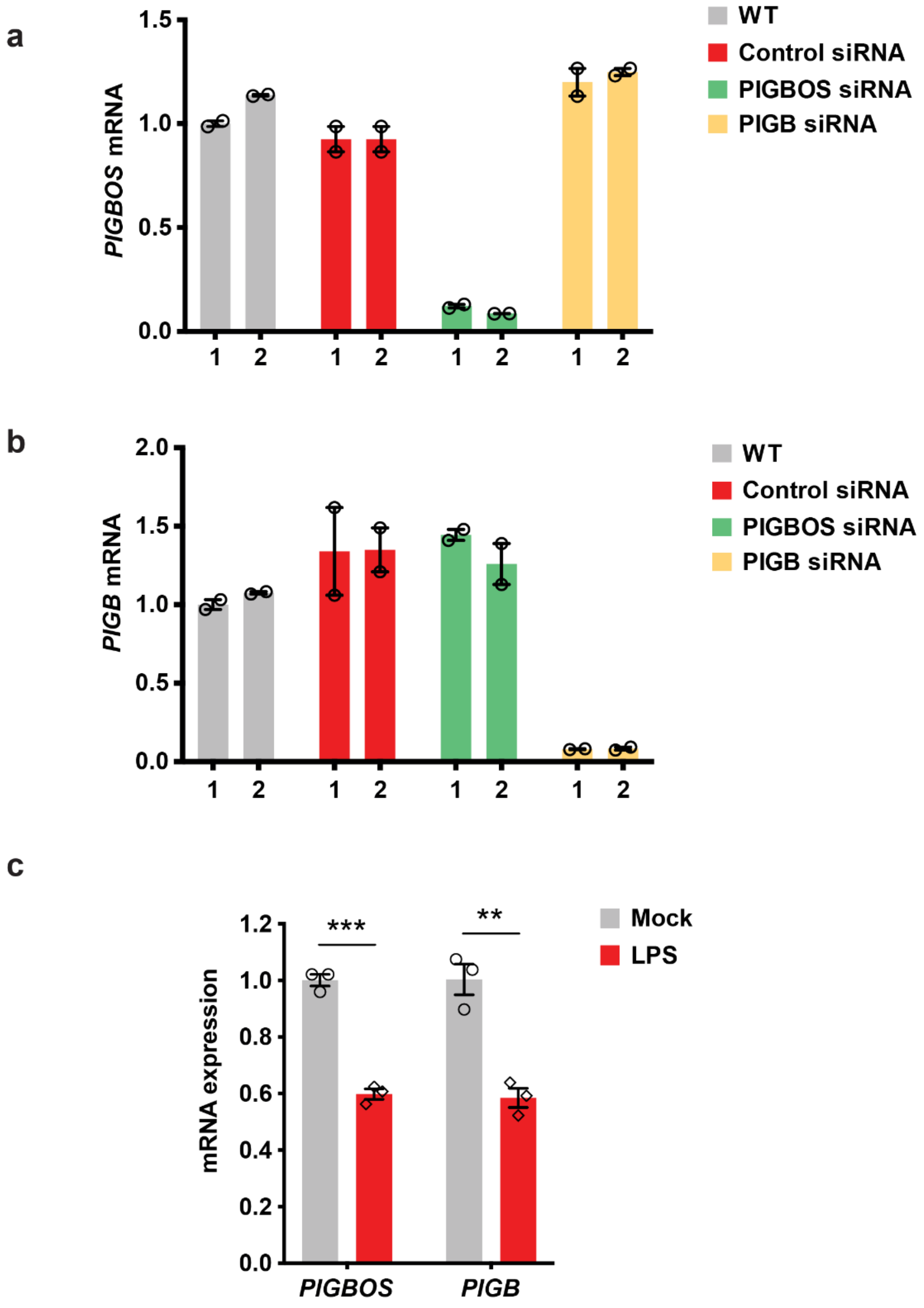

Supplementary Fig. 20. Regulation of PIGBOS and PIGB expression. a and **b**, HEK293 cells were transfected with indicated siRNA. *PIGBOS* and *PIGB* mRNA levels were quantified by RT-qPCR. Data were shown for two independent experiments, and errors (s.d.) were calculated based on two replicates for each condition. **c**, RAW 264.7 cells were treated with 100 ng/ml of LPS for 6 hours. *PIGBOS* and *PIGB* mRNA levels were measured by RT-qPCR. Error bars, s.e.m.. The *p*-values were calculated using Student's t-test, ***p* < 0.01, ****p* < 0.001, n = 3. Source Data are provided as a Source Data file.

Supplementary Fig. 21. Uncropped scans of Western blots in this study.

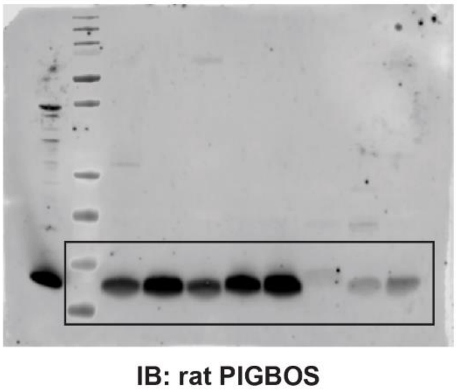

Figure 1c

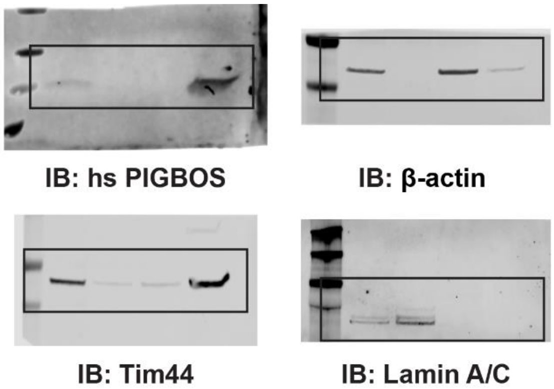

Figure 2a

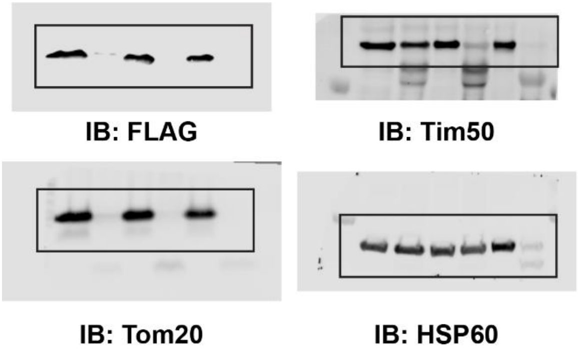

Figure 2c

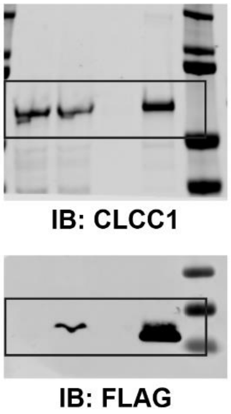

Figure 3b

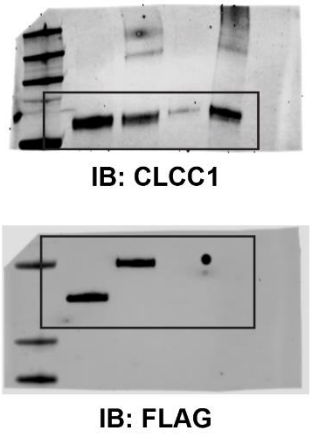

Figure 3d

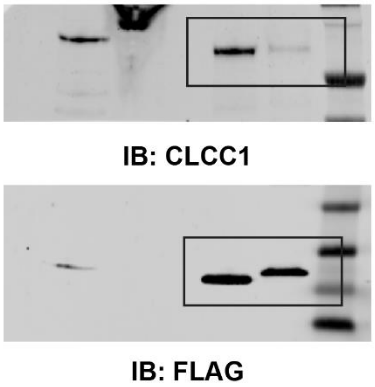

Figure 3e

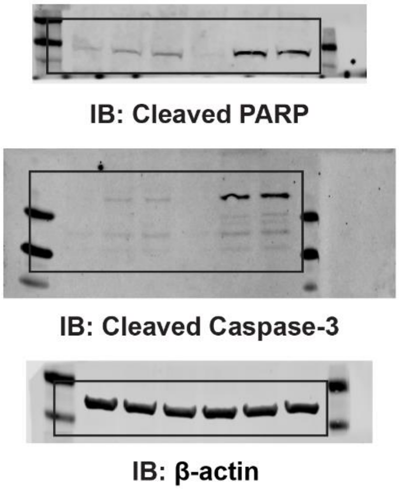

Figure 6f

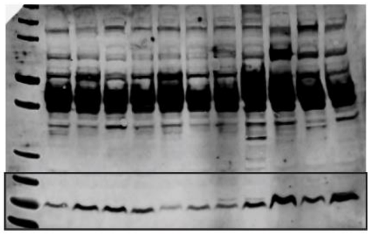

IB: hs PIGBOS

Supplementary Fig. 1b

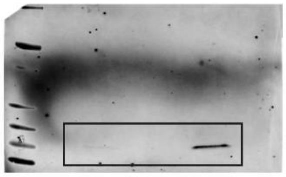

IB: FLAG

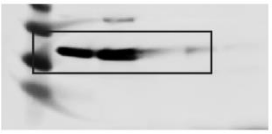

IB: Histone 2A

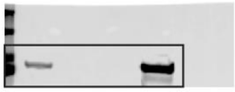

IB: mitofilin

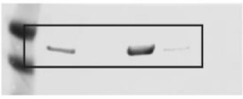

IB: β-actin

Supplementary Fig. 2a

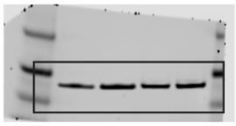

IB: Sec61b

Supplementary Fig. 3a

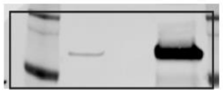

IB: HA

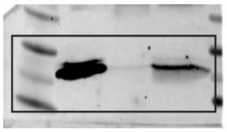

IB: FLAG

Supplementary Fig. 3b

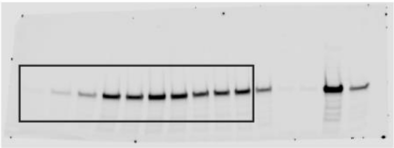

IB: CLCC1

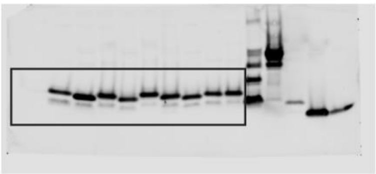

IB: FLAG

Supplementary Fig. 6c

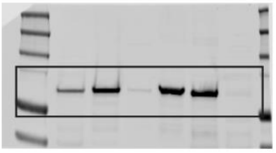

IB: CLCC1

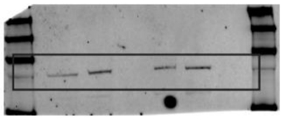

IB: Calnexin

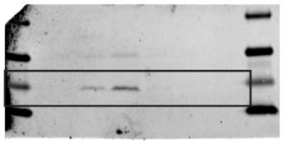

IB: PIGBOS

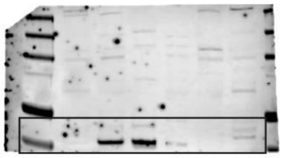

IB: Tim50

Supplementary Fig. 7a

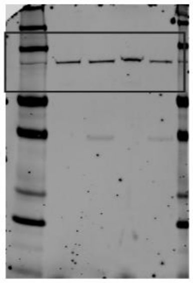

IB: Calnexin

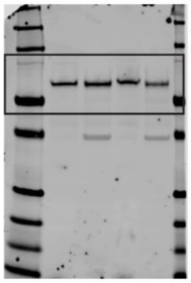

IB: CLCC1

Supplementary Fig. 7b

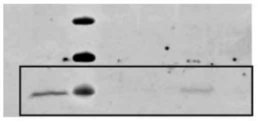

IB: hs PIGBOS

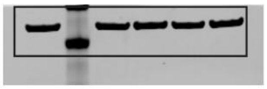

IB: β-actin

Supplementary Fig. 8b

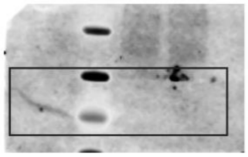

IB: hs PIGBOS

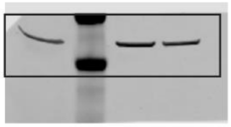

IB: β-actin

Supplementary Fig. 10c

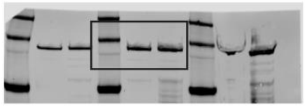

IB: HA

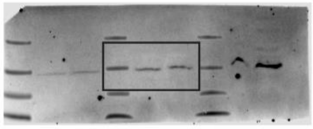

IB: FLAG

Supplementary Fig. 11b

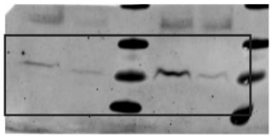

IB: hs PIGBOS

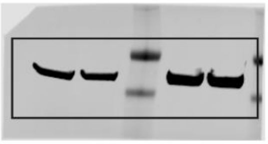

IB: β-actin

Supplementary Fig. 12b

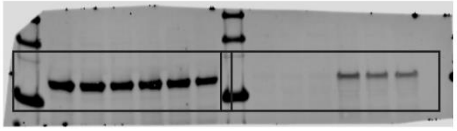

IB: CLCC1

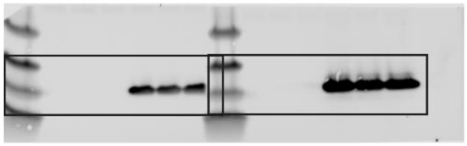

IB: FLAG

Supplementary Fig. 14a

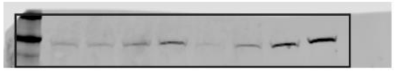

IB: cleaved PARP

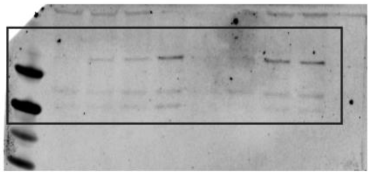

IB: cleaved Caspase-3

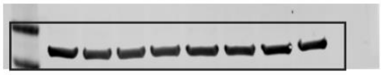

IB: β-actin

Supplementary Fig. 16b

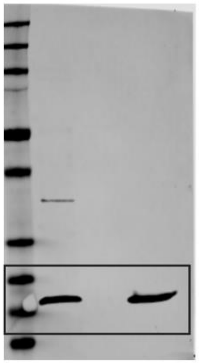

IB: FLAG

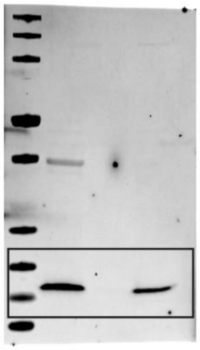

IB: HA

Supplementary Fig. 18b

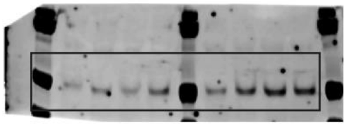

IB: ATF4

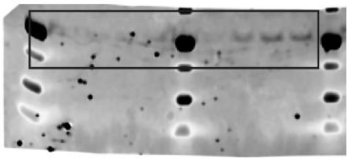

IB: CHOP

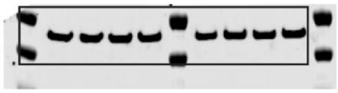

IB: β-actin

Supplementary Fig. 12h

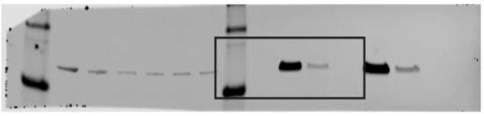

IB: CLCC1

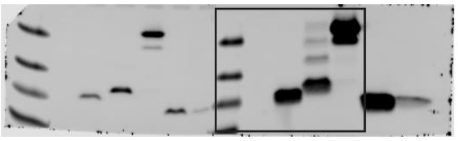

IB: FLAG

Supplementary Fig. 17e

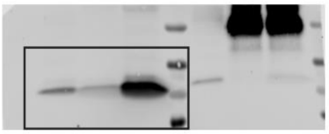

IB: HA

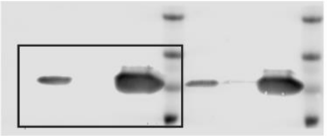

IB: FLAG

Supplementary Fig. 18c

Supplementary Table 1. List of PIGBOS interacting proteins after removal of false positives

No.	Bait	Prey	Prey Gene	Spec	SpecSum	Avg. Spec	Num Replicates	Ctrl. Counts	AvgP	MaxP	Topo AvgP	Topo MaxP	Saint Score	logOddsScore	FoldChange	BFDR
1	PIGBOS	P14625	ENPL	5|3|3	11	3.67	3	0|0|0	1	1	1	1	1	5.13	36.67	0
2	PIGBOS	Q96S66	CLCC1	49|27|29	105	35	3	2|0|0	1	1	1	1	1	15.92	52.5	0
3	PIGBOS	Q9NY65	TBA8	25|11|10	46	15.33	3	0|0|0	1	1	1	1	1	16.48	153.33	0
4	PIGBOS	P60228	EIF3E	11|6|3	20	6.67	3	1|0|0	0.99	1	0.99	1	0.99	3.33	20	0
5	PIGBOS	P22102	PUR2	7|2|2	11	3.67	3	0|0|0	0.98	1	0.98	1	0.98	3.28	36.67	0.01
6	PIGBOS	Q13838	DX39B	4|2|2	8	2.67	3	0|0|0	0.98	1	0.98	1	0.98	3.28	26.67	0.01
7	PIGBOS	P33993	MCM7	10|2|2	14	4.67	3	0|0|0	0.98	1	0.98	1	0.98	3.28	46.67	0
8	PIGBOS	O00148	DX39A	4|2|2	8	2.67	3	0|0|0	0.98	1	0.98	1	0.98	3.28	26.67	0.01
9	PIGBOS	Q86VP6	CAND1	9|3|2	14	4.67	3	1|0|0	0.91	1	0.91	1	0.91	1.13	14	0.01
10	PIGBOS	Q14152	EIF3A	7|3|2	12	4	3	1|0|0	0.91	1	0.91	1	0.91	1.13	12	0.02
11	PIGBOS	Q14204	DYHC1	16|3|6	25	8.33	3	1|1|0	0.9	1	0.9	1	0.9	0.91	12.5	0.03

Supplementary Table 2. List of PIGBOS interacting proteins after removal of false positives and contaminating proteins

No.	Bait	Prey	Prey Gene	Spec	SpecSum	Avg. Spec	Num Replicates	Ctrl. Counts	AvgP	MaxP	Topo AvgP	Topo MaxP	Saint Score	logOddsScore	FoldChange	BFDR
1	PIGBOS	Q96S66	CLCC1	49|27|29	105	35	3	2|0|0	1	1	1	1	1	15.92	52.5	0

Supplementary Table 3. List of siRNA used in this study

Name	Information
PIGBOS1 siRNA SMARTpool	Dharmacon R-192024-00-0005
PIGBOS1 individual siRNA (For PIGBOS-FLAG rescue experiments)	Dharmacon N-192024-01-0002
CLCC1 siRNA SMARTpool	Dharmacon L-010024-00-0005
Negative control non-targeting siRNA	Life Technologies 12935-113

Supplementary Table 4. List of sequences of RT-qPCR primers

**Name**	**Sequence (5’->3’)**
*PIGBOS1*_f	TGTTGGTGTTAACATTTCAGATTT
*PIGBOS1*_r	GGACAGTGGCAAAAAGCAGT
*GRP78*_f	GCCTGTATTTCTAGACCTGCC
*GRP78*_r	TTCATCTTGCCAGCCAGTTG
*CHOP*_f	GGAGCATCAGTCCCCCACTT
*CHOP*_r	TGTGGGATTGAGGGTCACATC
*Ero1LB*_f	TTCTGGATGATTGCTTGTGTGAT
*Ero1LB*_r	GGTCGCTTCAGATTAACCTTGT
*ERdj4*_f	GGAAGGAGGAGCGCTAGGTC
*ERdj4*_r	ATCCTGCACCCTCCGACTAC
*Edem*_f	TTCCCTCCTGGTGGAATTTG
*Edem*_r	AGGCCACTCTGCTTTCCAAC
*Hyou*_f	GCAGACCTGTTGGCACTGAG
*Hyou*_r	TCACGATCACCGGTGTTTTC
*GAPDH*_f	CATGTTCCAATATGATTCCACC
*GAPDH*_r	CTCCACGACGTACTCAGCG
*XBP1s*_f	CTGAGTCCGCAGCAGGTG
*XBP1s*_r	GTCCAGAATGCCCAACAGGA
*HSPD1*_f	CTACTGTACTGGCACGCTCTA
*HSPD1*_r	CAACAGCTAACATCACACCTCTC

Supplementary Table 5. List of sequences of gRNA protospacers and genotyping primers

**Protospacer**	**Sequence (5'->3')**
PIGBOS1 gRNA-1	AGTTAATACTACATGGAGTT
PIGBOS1 gRNA-2	GCCACTGTCCTTGGAATTGC
PIGBOS1 gRNA-3	CGAGTTTAGTGACGTGGAGC
**Genotyping primers**	**Sequence (5'->3')**
P3	CCCTTCCCTAATTCACTTCCT
P4	TCCTTCTGATCTTTGGCATACTG
P5	CCTGGTAAAAAGACATAAAATGACA

Supplementary Table 6. List of antibodies used in this study

**Primary antibody**	**Company**	**Catalog number**	**Dilution**
FLAG	Sigma	F1804	1:1000
DYKDDDDK Tag	Cell Signaling	2368	1:1000
CLCC1	Sigma	HPA009087	1:1000
ATF4	Cell Signaling	11815S	1:1000
CHOP	Cell Signaling	2895S	1:1000
Cleaved PARP	Cell Signaling	5625S	1:1000
beta-Actin	LiCor	926-42210	1:1000
HA	Sigma	H9658	1:1000
HA-Tag	Cell Signaling	3724	1:1000
Histone H2A	Cell Signaling	12349S	1:1000
Tom20	Santa Cruz Biotechnology	sc-11415	1:1000
Tim50	Abcam	Ab23938	1:1000
HSP60	Abcam	Ab46798	1:1000
Mitofilin	Abcam	Ab93323	1:1000
Cleaved Caspase-3	Cell Signaling	9661S	1:1000
Lamin A/C	Cell Signaling	2032S	1:1000
Tim44	Atlas Antibodies	HPA043052	1:1000
Calnexin	Cell Signaling	2679S	1:1000
Sec61b	Cell Signaling	14648	1:1000
GM130	Cell Signaling	12480	1:1000
**2nd antibody**			
IRDye 800CW Goat anti-mouse	LiCor	926-32210	1:10000
IRDye 800CW Goat anti-Rabbit	LiCor	926-32211	1:10000
IRDye 800CW Donkey anti-Goat	LiCor	926-32214	1:10000
IRDye 800CW Donkey anti-guinea pig	LiCor	925-32411	1:10000
Alexa Fluor 488 goat anti mouse IgG	Life Technologies	A11001	1:1000
Alexa Fluor 546 goat anti mouse IgG	Life Technologies	A11003	1:1000
Alexa Fluor 546 goat anti rabbit IgG	Life Technologies	A11010	1:1000
Alexa Fluor 647 goat anti rabbit IgG	Life Technologies	A21245	1:1000

Supplementary Table 7. List of DNA constructs used in this study

Name	Features	Vector/promoter	Notes
PIGBOS-FLAG	PIGBOS-linker-FLAG	pcDNA3.1(+)/CMV	Linker: GGS<br>FLAG: DYKDDDDK
APEX	FLAG-APEX2	pcDNA3/CMV	Originally from A. Ting pcDNA3-APEX2-NES (Addgene #49386). Insert a stop codon before NES
PIGBOS-APEX	FLAG-PIGBOS-linker-APEX-myc	pcDNA3.1(+)/CMV	FLAG: DYKDDDDK<br>Linker: GSGSGSTSGSG
PIGBOS-3×GFP11-FLAG	PIGBOS-3×GFP11-FLAG	pcDNA3.1(+)/CMV	3×GFP11 sequence was adopted from Nat. Comm. 2016, 7, 11046.
3×GFP11-PIGBOS-FLAG	3×GFP11-PIGBOS-FLAG	pcDNA3.1(+)/CMV	3×GFP11 sequence was adopted from Nat. Comm. 2016, 7, 11046.
PIGBOS-ΔC-3×GFP11-FLAG	PIGBOS(1-29)- 3×GFP11-FLAG	pcDNA3.1(+)/CMV	3×GFP11 sequence was adopted from Nat. Comm. 2016, 7, 11046.
GFP(1-10)	GFP (1-10)	pcDNA3.1(+)/CMV	This construct was a gift from Bo Huang (Addgene #70219).
CLCC1-HA	CLCC1-linker-HA	pcDNA3.1(+)/CMV	Linker: GSGSTS<br>GFP(1-10) sequence was PCR amplified from Addgene #70219
CLCC1-GFP(1-10)	CLCC1-linker-GFP(1-10)-HA	pcDNA3.1(+)/CMV	Linker: GGS<br>HA: YPYDVPDYA
Sec61b-mCherry	mCherry-Sec61 beta	Modified pAcGFP1-C1/CMV	This construct was a gift from Gia Voeltz (Addgene #49155).
p5xATF6-GL3	5xATF6 binding site	pOFluc-GL3	This construct was a gift from Ron Prywes (Addgene #11976)
PIGBOS gRNA-1	PIGBOS gRNA-1	px458-mCherry/U6	Replace GFP in px458 to mCherry, subclone gRNA-1 between BbsI sites
PIGBOS gRNA-2	PIGBOS gRNA-2	px458-GFP/U6	subclone gRNA-2 between BbsI sites
PIGBOS gRNA-3	PIGBOS gRNA-3	px458-GFP/U6	Subclone gRNA-3 between BbsI sites
HA-VAPB	HA-linker-VAPB	pcDNA3.1(+)/CMV	Linker: GSGSGSTS<br>HA: YPYDVPDYA
PTPIP51-FLAG	PTPIP51-linker-FLAG	pcDNA3.1(+)/CMV	Linker: GGSGGGGS<br>FLAG: DYKDDDDK
Tom20-PIGBOS(30-54)	Tom20-PIGBOS(30-54)-FLAG	pcDNA3.1(+)/CMV	PIGBOS cytosolic domain (30-54) is appended at the C-term of full-length Tom20
C1-PIGBOS(30-54)	C1(1-27)-PIGBOS(30-54)-FLAG	pcDNA3.1(+)/CMV	C1(1-27) represents the first 27 aa from rabbit cytochrome P450 2C1
PIGBOS AAA mutants	Triple alanine scan of PIGBOS cytosolic domain (30-54)	pcDNA3.1(+)/CMV	Triple alanine (AAA) mutants was introduced as indicated in the PIGBOS-3×GFP11-FLAG

Supplementary Table 8. List of PCR primers used in this study

Construct	Note	Primer	Sequence
PIGBOS-APEX	two fragments overlapping PCR, insert into pcDNA3.1(+)	1_forward	gatcGGATCCATGGACTACAAGGATGACGACGATAAGTTTAGGAGATTGACTTTTGCAC
		1_reverse	GCCCGAGCCCGAGGTCGAGCCCGAGCCACTTCCTCCACTTTTCTTCTC
		2_forward	GGCTCGGGCTCGACCTCGGGCTCGGGCGGAAAGTCTTACCCAACTG
		2_reverse	gtatctcgagTTACAGATCCTCTTCTGAGATGAGTTTTTGTTCGGCATCAGCAAACCC
3xGFP11-PIGBOS-FLAG	Insert 3xGFP11 sequence in PIGBOS-FLAG construct	forward	atcgGGTACCATGCGTGACCACATGGTCCT
		reverse	tgcaGGATCCTCCAGAGCCACCGGTTATTCCGGCTGCATTG
PIGBOS-ΔC-3xGFP11-FLAG	remove C-Term of PIGBOS by Q5 mutagenesis	forward	agacgatgacgacaagTAACTCGAGTCTAGAGGG
		reverse	ttgtagtcacttcctccGGTTATTCCGGCTGCATT
PIGBOS AAA mutants	mutate corresponding aa to alanine by Q5 mutagenesis	forward	GGCTGCCAAAGATCAGAAGGAATTAAAAG
	30-32 AAA	reverse	GCTGCAAATACTGGTTGAAAAATATATACTCc
	34-36 AAA	forward	agcgAAGGAATTAAAAGAAAAGATGC
		reverse	gcagcGGCATACTGTTCAAATAC
	37-39 AAA	forward	CAGCAAAAGAAAAGATGCAGTTGGtacaag
		reverse	CCGCCTGATCTTTGGCATACTGttcaaatactgg
	40-42 AAA	forward	CAGCGATGCAGTTGGTACAAGAATCagaagag
		reverse	CGGCTAATTCCTTCTGATCTTTGGCatactg
	43-45 AAA	forward	GGCAGTACAAGAATCAGAAGAGAAGaaaagtgg
		reverse	GCAGCCTTTTCTTTTAATTCCTTCTGATCtttgg
	46-48 AAA	forward	ggcaTCAGAAGAGAAGAAAAGTG
		reverse	GCAGCCAACTGCATCTTTTCTTTTAATTCcttctg
	49-51 AAA	forward	CTGCGAAGAAAAGTGGTGGCTCTG
		reverse	CTGCTTCTTGTACCAACTGCATC
	52-54 AAA	forward	cagctGGTGGCTCTGGAGGTCGT
		reverse	CCGCCTCTTCTGATTCTTGTACCAACtgc
CLCC1-GFP(1-10)	Insert CLCC1 into the GFP(1-10) construct	forward	gatcGAATTCatgctgtgttctttgctcctttg
		reverse	CAACACCGGTAAACAGTTCTTCTCCTTTGGACGAGGTCGAGCCCGAGCCgccacaggggctgctgaccgg
	Add HA tag after EGFP by Q5 mutagenesis	forward	TACGATGTTCCAGATTACGCTTAGTCTAGAGGGCCCGTTTAAACC
		reverse	TGGGTATCCTCCACTACCGCCACTTCCGCCGCCACCTGT
HA-GFP(1-10)-VAPB	two fragments overlapping PCR, insert into pcDNA3.1(+)	1_forward	gtcaggatccatgTACCCATACGATGTTCCAGATTACGCTTCCAAAGGAGAAGAACTGTT
		1_reverse	ttcgcCGAGGTCGAGCCCGAGCCACTTCCGCCGCCACCTGTT
		2_forward	GAAGTGGCTCGGGCTCGACCTCGgcgaaggtggagcaggtcctga
		2_reverse	gactTCTAGActacaaggcaatcttcccaataattac
HA-VAPB	remove GFP(1-10) by Q5 mutagenesis	forward	GAAGTGGCTCGGGCTCG
		reverse	CAGCGTAATCTGGAACATCGTATGG
PTPIP51-3xGFP11-FLAG	two fragments overlapping PCR, insert into pcDNA3.1(+)	1_forward	ctagggatccatgtctagactgggagccctggg
		1_reverse	acctccagagccaccgtctcgtaaaatgacttccagttct
		2_forward	gtcattttacgagacggtggctctggaggtcgtgacca
		2_reverse	gatcctcgagTTACTTGTCGTCATCGTCTTTGTAGTCAC
PTPIP51-FLAG	remove 3xGFP11 by Q5 mutagenesis	forward	GGAGGAAGTGACTACAAAGACG
		reverse	ACCTCCAGAGCCACCGTC
