# Supplementary material for: Regulation of the ER stress response by a mitochondrial microprotein
Source: Nat Commun. 2019 Oct 25;10:4883. doi: 10.1038/s41467-019-12816-z (PMC6814811; doi:10.1038/s41467-019-12816-z)
Supplement: Supplementary file 2 — Description of Additional Supplementary Files [file 41467_2019_12816_MOESM2_ESM.pdf]

Description of Additional Supplementary Files

File name: Supplementary Data 1

Description: List of PIGBOS interacting proteins from PIGBOS-FLAG IP-MS
